# Caspase-1 and Gasdermin D Afford the Optimal Targets with Distinct Switching Strategies in NLRP1b Inflammasome-Induced Cell Death

**DOI:** 10.34133/2022/9838341

**Published:** 2022-07-19

**Authors:** Xiang Li, Peipei Zhang, Zhiyong Yin, Fei Xu, Zhang-Hua Yang, Jun Jin, Jing Qu, Zhilong Liu, Hong Qi, Chenggui Yao, Jianwei Shuai

**Affiliations:** ^1^Department of Physics and Fujian Provincial Key Laboratory for Soft Functional Materials Research, Xiamen University, Xiamen 361005, China; ^2^National Institute for Data Science in Health and Medicine and State Key Laboratory of Cellular Stress Biology, Innovation Center for Cell Signaling Network, School of Life Sciences, Xiamen University, Xiamen 361102, China; ^3^Complex Systems Research Center, Shanxi University, Shanxi, Taiyuan 030006, China; ^4^College of Data Science, Jiaxing University, Jiaxing 314000, China; ^5^Oujiang Laboratory (Zhejiang Lab for Regenerative Medicine, Vision and Brain Health) and Wenzhou Institute, University of Chinese Academy of Sciences, Wenzhou, Zhejiang 325001, China

## Abstract

Inflammasomes are essential complexes of innate immune system, which form the first line of host defense against pathogens. Mounting evidence accumulates that inflammasome signaling is highly correlated with coronavirus disease 2019 (COVID-19). However, there remains a significant gap in our understanding of the regulatory mechanism of inflammasome signaling. Combining mathematical modeling with experimental analysis of NLRP1b inflammasome signaling, we found that only the expression levels of caspase-1 and GSDMD have the potential to individually switch cell death modes. Reduction of caspase-1 or GSDMD switches cell death from pyroptosis to apoptosis. Caspase-1 and GSDMD present different thresholds and exert distinct pathway choices in switching death modes. Pyroptosis switches to apoptosis with an extremely low threshold level of caspase-1, but with a high threshold of GSDMD. Caspase-1-impaired cells employ ASC-caspase-8-dependent pathway for apoptosis, while GSDMD-impaired cells primarily utilize caspase-1-dependent pathway. Additionally, caspase-1 and GSDMD can severally ignite the cooccurrence of pyroptosis and apoptosis. Landscape topography unravels that the cooccurrence is dramatically different in caspase-1- and GSDMD-impaired cells. Besides pyroptosis state and apoptosis state, a potential new “coexisting” state in single cells is proposed when GSDMD acts as the driving force of the landscape. The “seesaw model” is therefore proposed, which can well describe the death states that are controlled by caspase-1 or GSDMD in single cells. Our study sheds new light on NLRP1b inflammasome signaling and uncovers the switching mechanisms among various death modes, providing potential clues to guide the development of more rational control strategies for diseases.

## 1. Introduction

Inflammasomes are multiprotein complexes that shape host immune responses to invading pathogens and infections but can also lead to sepsis and host death if overactivated [1, 2]. The immune defense mechanism is initiated through the activation of pattern recognition receptors (PRRs) in response to pathogen-associated molecular patterns (PAMPs) or endogenous danger-associated molecular patterns (DAMPs). Inflammasome functions as a platform for the activation of caspase-1, which subsequently cleaves gasdermin D (GSDMD) into N-terminal (N-GSDMD) and C-terminal (C-GSDMD) [3–5]. The cleaved N-GSDMD fragment can form pores in the membrane that lead to pyroptosis, resulting in the secretion of proinflammatory cytokines such as interleukin-1*β* (IL-1*β*) and interleukin-18 (IL-18) [6–8]. There are four well-known canonical inflammasome branches, including NLRP3 (NACHT, leucine-rich repeat, and pyrin domain- (PYD-) containing protein 3), NLRP1, NLRC4 (NLR family caspase recruitment domain- (CARD-) containing protein 4), and absent in melanoma-2 (AIM2) [2]. NLRP3 and AIM2 can interact with adaptor protein ASC through the N-terminal PYD domain, and then, the CARD domain of ASC recruits caspase-1 to activate it and mediate pyroptosis. In the case of NLRC4 and murine NLRP1b, they can recruit ASC directly through their CARD and subsequently activate caspase-1. Besides, NLRC4 and NLRP1b can also directly interact with caspase-1 via their CARD to mediate pyroptosis in an ASC-independent manner [2, 9].

Aberrant inflammasome signaling is associated with multiple autoimmune diseases, neurodegeneration, metabolic disorders, Alzheimer's disease, and cancer [10]. Pyroptosis exerts a significant role in the clearance of infectious agents by releasing the surviving intracellular bacteria for neutrophil-mediated killing, whereas pyroptosis also leads to the pathology of various diseases through triggering strong inflammatory responses [11]. More importantly, mounting evidence is now accumulating that SARS-CoV-2 can directly or indirectly activate inflammasomes, playing critical role in severe COVID-19 [12–14]. As an upstream of cytokine release, pyroptosis signaling is an attractive target for inflammatory diseases. A recent study indicates that up to 63% of people infected with COVID-19 are highly correlated with pyroptosis signaling [15]. Pyroptosis signaling inhibitors exert great function on COVID-19 treatment. The inhibitors, such as chloroquine and hydroxychloroquine that effectively block the activation of inflammsome induced by SARS-CoV-2, have been applied in COVID-19 treatment [16]. Besides, IL-1 antagonist (anakinra), which blocks the proinflammatory effect of IL-1, has successfully treated a COVID-19 patient [17]. Thus, a better understanding of inflammasome signaling regulatory mechanism enables the potential novel therapeutic strategies for various diseases.

Recent studies suggested that inflammasomes induce not only pyroptosis but also apoptosis [3, 9, 18]. In contrast to the various diseases triggered by pyroptosis, apoptosis is a programmed cell death mode that avoids eliciting inflammation. Apoptosis is essential and confers advantages for cellular development, homeostasis, and disease prevention [19]. Accumulating evidence suggests that targeting apoptosis signaling might be also a promising strategy in COVID-19 treatment [20, 21]. Our latest study shows that there are three cell death pathways of pyroptosis and apoptosis downstream of inflammasome activation [9]. Nevertheless, the fundamental questions how the connectivity of the three pathways generates specific cell fate and what are their potential switching mechanisms remain unclear. To systematically explore the underlying mechanisms of the newly identified pathways, analysis that combined mathematical modeling with quantitative western blot is employed based on the NLRP1b inflammasome signaling, which was well established in our previous study [9]. We identified that among all the NLRP1b-inflammasome signaling transducers, only caspase-1 and GSDMD have the potential to individually switch cell death modes between pyroptosis and apoptosis. Caspase-1 and GSDMD present different thresholds and exert distinct pathway choices in determining cell death modes. Caspase-1 and GSDMD are found that can severally ignite the cooccurrence of pyroptosis and apoptosis, while the cell death landscape topographies strikingly suggest two potential distinct cooccurrence death modes. These results unveil several quantitative new insights into the inflammasome-induced cell death signaling, providing potential therapeutic strategies for controlling various death modes.

## 2. Results

### 2.1. Quantification of the Three Death Pathways Activated by NLRP1b Inflammasome

**Figure 1:**
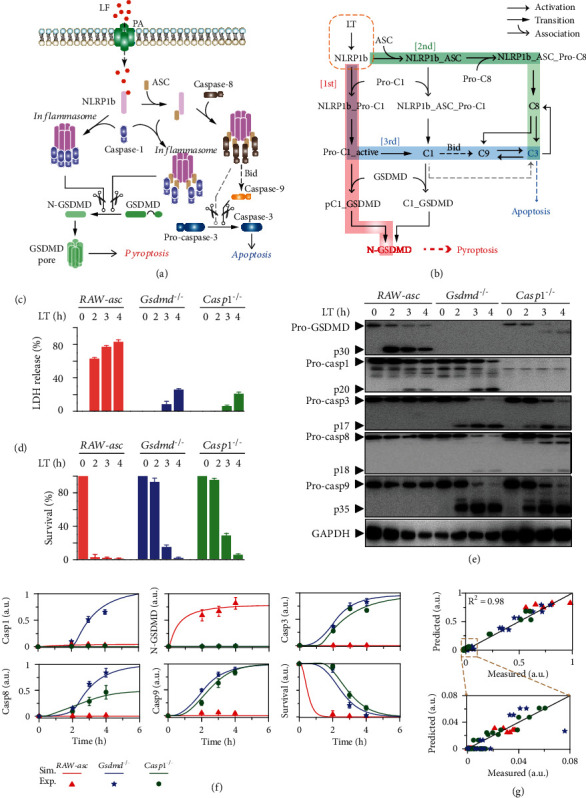
Data-driven modeling of the three cell death pathways induced by NLRP1b inflammasome. (a) Schematic diagram of lethal toxin (LT) induced NLRP1b inflammasomes assembly and downstream signaling activation. (b) Kinetic scheme of the three newly identified death pathways induced by NLRP1b inflammasome activation. The dashed lines labelled with Bid indicate the indirect activation of caspase-9 by caspase-1 through the intrinsic apoptosis pathway. The gray dashed lines describe the direct slight cleavage of caspase-3 by caspase-1. (c, d) Effects of genetic deletion of *Gsdmd* or *Casp1* on inflammasome-induced LDH release and cell survival for indicated times. (e) Western blot analysis of the effects of deletion of *Gsdmd* or *Casp1* on indicated protein activation. (f) Comparison between experimental data (dots) and simulation results (lines) in *RAW-asc*, *Gsdmd^−/−^*, and *Casp1^−/−^* cells. (g) Scatter diagram of experimental data versus simulation results. *R*^2^ represents the deviation between experimental data and simulation results.

The NLRP1b inflammasome activation-induced cell death can be generally illustrated by the signaling transducers shown in Figure 1(a). Upon lethal toxin (LT: lethal factor plus PA) stimulation, NLRP1b can directly recruit caspase-1 through its CARD domain to form inflammasome [22]. PA means anthrax protective antigen in this study. LF (lethal factor) can enter cells by PA-mediated endocytosis, and the cytoplasmic LF leads to NLRP1b-dependent pyroptosis [23]. The direct recruitment of caspase-1 by NLRP1b does not result in the proteolysis of caspase-1. Besides, NLRP1b can also bind to ASC through the PYD domain, and ASC further recruits caspase-1, resulting in the formation of inflammasome [24]. In this case, ASC is required, and caspase-1 is activated in inflammasome by autocatalytic cleavage. The inflammasomes present a speck-like oligomeric structure. In different inflammasomes, both the uncleaved and cleaved caspase-1 can in turn cleave GSDMD [3, 25]. The N-terminal domain of the cleaved GSDMD (N-GSDMD) forms pore structure in the membrane to induce pyroptosis. When pyroptosis is blocked, NLRP1b-ASC complex can also recruit caspase-8, initiating the cleavage of caspase-3 and apoptosis [3, 26].

To fully understand inflammasome-induced cell death, we recently showed that there are three death pathways which complement each other [9]. The first/default path is the caspase-1 and GSDMD-mediated pyroptosis pathway (Figure 1(b), red background). The second path is the ASC and caspase-8-mediated apoptosis pathway when pyroptosis is impaired (Figure 1(b), green background). Double knockout of *Gsdmd* and *Asc* cannot block cell death, which is contributed by the third/intrinsic apoptosis pathway mediated by caspase-1 (Figure 1(b), blue background). The caspase-1-induced intrinsic apoptosis pathway involves the Bid-Apaf-1-caspase-9-caspase-3 axis [18, 27], which is also confirmed by using *Gsdmd^−/−^Casp8^−/−^*, *Gsdmd^−/−^Casp8^−/−^Casp1^−/−^*, *Gsdmd^−/−^Casp8^−/−^Apaf1^−/−^*, and *Gsdmd^−/−^Casp8^−/−^Casp9^−/−^* cells in our recent study [9]. Moreover, the caspase-1 recruited by NLRP1b-ASC complex can also induce the occurrence of pyroptosis via cleaving GSDMD [26]. Cleaved caspase-1 was recently reported that could directly activate caspase-3 [28]. However, previous studies suggested that the strength of the direct cleavage of caspase-3 by caspase-1 seems to be quite weak in RAW-asc cells [9, 18]. Meanwhile, cleaved caspase-3 also activates caspase-8/9, providing efficient positive feedback for apoptosis induction [29].

To quantitatively investigate dynamic of the transducers and their functions in cell death decision-making, experimental analysis was first performed to obtain the pathway responses upon stimulation (Figures 1(c)–1(e)). RAW-asc, a RAW264.7-derived cell line containing ectopically expressed ASC, is used to observe the time responses of the transducers. Occurrence of pyroptosis is measured by the release of lactate dehydrogenase (LDH) (Figure 1(c)). Compared to RAW-asc cells, deletion of *Gsdmd* or *Casp1* effectively blocks LDH release at 2 hours, but LDH is still released at later times due to the induction of secondary necrotic lysis [30, 31]. Besides, deletion of *Gsdmd* or *Casp1* exhibits similar patterns in inhibiting cell death at 2-hour LT treatment (Figure 1(d)). The cell death mode switching from pyroptosis to apoptosis is further determined by measuring the time series of the key transducer activities (Figure 1(e)). Western blotting analysis indicates that only the activities of GSDMD (p30) and caspase-1 (p20) can be detected, while the apoptotic transducers caspase-8/9/3 (p18/p35/p17) remain inactive in RAW-asc cells. Pyroptosis occurs under this condition. In contrast, activation of caspase-8/9/3 is detected in *Gsdmd^−/−^* or *Casp1^−/−^* cells, indicating the switch of pyroptosis to apoptosis. Caspase-9/3 are cleaved in both *Gsdmd^−/−^* and *Casp1^−/−^* cells, whereas the activation of caspase-1 is significantly increased in *Gsdmd^−/−^* cells, implying the potential different switch mechanisms of death mode in *Gsdmd^−/−^* and *Casp1^−/−^* cells. Similar results are observed in J774A.1 cell line (Figure S1), suggesting that the switch mechanisms in inflammasome activation might be generally applicable.

Based on the three identified pathways (Figure 1(b)), we developed a corresponding mathematical model, which incorporates transducer association/disassociation, activation, cleavage, and enzymatic reactions. A complete schematic diagram of the biochemical reactions in the NLRP1b inflammasome signaling can be found in Figure S2. These biochemical reactions are represented by molecule-molecule interactions and enzymatic reactions, and the reaction rates are dependent on protein amounts and kinetic rate constants according to the law of mass action. All the corresponding reactions in Figure S2 and reaction rates in model are elaborated in Table S1. Using the well-established kinetic approaches [32, 33], these biochemical processes can be subsequently translated into a set of ODEs (ordinary differential equations) to describe the time evolution of amount for each molecular species (Table S2). Initial amount of the molecular species is estimated from experimental studies [34–36]. The kinetic parameters are reasonably estimated with biochemical constraints [32] and are mostly determined by a global optimization method that minimizes the deviation between simulation results and experimental data (Table S3). The caspase-1-induced intrinsic apoptosis pathway involves the Bid-Apaf-1-caspase-9-caspase-3 axis, which is a complicated process [18, 27]. Considering these reactions in model needs dozens of ODEs, and some complicated processes such as the release of contents from mitochondrion will make the model very complex; we therefore simplified the intrinsic apoptosis pathway by sketchily modeling the relation between caspase-1 and caspase-9 to focus on exploring the inflammasome-induced cell death switch in this study. Comparison results indicate that the model can well reproduce the time responses of the transducers under different conditions (Figure 1(f)). Significantly, the normalized blotting data shown in Figure 1(f) suggests that caspase-8 activation in *Gsdmd^−/−^* is two times higher than that in *Casp1^−/−^* cells, hinting the potential different apoptosis pathways in these cells. The comparison results can be reflected clearly by the statistical chart shown in Figure 1(g), confirming that our model has the potential for giving mechanistic insights and exploring the emergent properties of inflammasome in death decision-making.

### 2.2. Multiple Knockout Analysis Authenticates the Induction of Two Distinct Apoptosis Pathways

**Figure 2:**
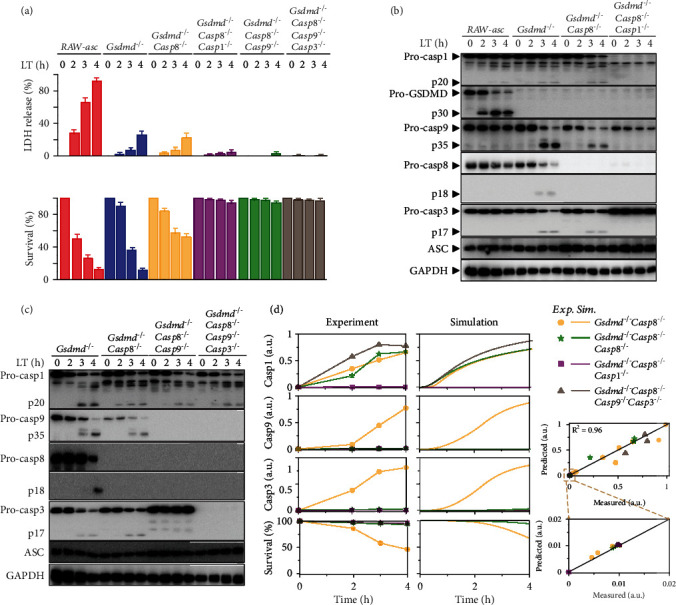
Effects of multiple knockout of proteins on cell death pathways. (a–c) Effects of *Gsdmd/Casp8* double knockout, *Gsdmd/Casp8/Casp1* triple knockout, *Gsdmd/Casp8/Casp9* triple knockout, and *Gsdmd/Casp8/Casp9/Casp3* quadruple knockout on LDH release, cell survival rate, and protein activation for indicated times. (d) Comparison between experimental data (dots) and simulation results (lines) in the multiple knockout cells.

As the normalized blotting data indicates that caspase-9/3 activation is similar, while caspase-8 is different in *Gsdmd^−/−^* and *Casp1^−/−^* cells (Figure 1(f)), we further tested the caspase functions in cell death of multiple knockout cells. Time responses of LT-induced LDH release and cell death are measured in *Gsdmd^−/−^Casp8^−/−^*, *Gsdmd^−/−^Casp8^−/−^Casp1^−/−^*, *Gsdmd^−/−^Casp8^−/−^Casp9^−/−^*, and *Gsdmd^−/−^Casp8^−/−^Casp9^−/−^Casp3^−/−^* cell lines (Figure 2(a)). Deletion of *Gsdmd* blocks LDH release and induces apoptosis, which occur in *Gsdmd^−/−^Casp8^−/−^* cells as well, proving that the induction of apoptosis in *Gsdmd^−/−^* cells can bypass caspase-8. Caspase-8 indeed participates in promoting apoptosis in *Gsdmd^−/−^* cells since the death rate decreases in *Gsdmd^−/−^Casp8^−/−^* cells. However, either *Gsdmd^−/−^Casp8^−/−^Casp1^−/−^* or *Gsdmd^−/−^Casp8^−/−^Casp9^−/−^* cells are resistant to cell death, suggesting that both caspase-1 and caspase-9 are essential for GSDMD deletion-induced apoptosis.

Time responses of the key transducers in the multiple knockout cells are further evaluated by western blotting analysis. Compared to *Gsdmd^−/−^*, caspase-9 activation is also detected in *Gsdmd^−/−^Casp8^−/−^*, but completely blocked in *Gsdmd^−/−^Casp8^−/−^Casp1^−/−^* cells, confirming that caspase-8 is not essential but caspase-1 is required for caspase-9 activation (Figure 2(b)). Caspase-9/3 activation occur in either *Casp1^−/−^* (Figure 1(e)) or *Gsdmd^−/−^Casp8^−/−^* but are completely blocked in *Gsdmd^−/−^Casp8^−/−^Casp1^−/−^* cells (Figure 2(b)). These results validate the 2nd and 3rd apoptosis pathways shown in Figure 1(b) that at least one of them (i.e., caspase-1 and caspase-8) is essential for caspase-9/3 activation and apoptosis induction. Besides, caspase-3 activation occurs in either *Casp1^−/−^* (Figure 1(e)) or *Gsdmd^−/−^Casp8^−/−^* (Figure 2(b)) but is blocked in *Gsdmd^−/−^Casp8^−/−^Casp9^−/−^* cells (Figure 2(c)), proving that at least one of them (caspase-8 and caspase-9) is required for apoptosis induction.

To examine the reliability of our mathematical model, the corresponding transducer dynamics and cell death under multiple knockout are simulated. Comparison between the experimental data and simulation results is shown in Figure 2(d), indicating that the multiple knockout cases predicted by our model are also quantitatively supported by experiments. Although caspase-3 activation can hardly be detected (Figure 2(c)), cell death seems to slightly occur after 4-hour treatment in *Gsdmd^−/−^Casp8^−/−^Casp9^−/−^* cells (Figure 2(a)), implying the potential mechanism of caspase-1-induced direct activation of caspase-3. However, both our experimental data and simulation results indicate that the direct cleavage cannot result in an obviously increase of caspase-3 activity, which is consistent with the previous observations [9, 18]. As a result, the high consistency between simulation and experiment (Figures 1(g) and 2(d)) affirms that our model has high confidence for further clarifying the switching mechanisms within the NLRP1b-inflammasome-induced three cell death pathways.

### 2.3. Caspase-1 and GSDMD Are the Only Switches between Pyroptosis and Apoptosis

**Figure 3:**
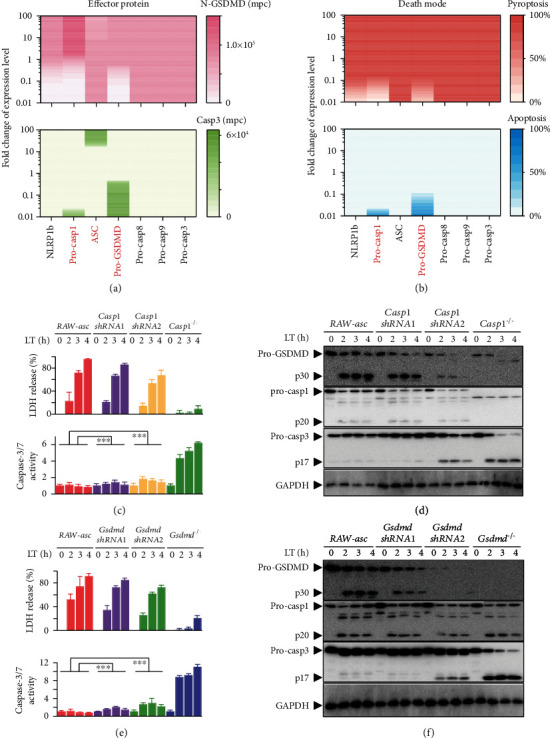
Roles of the NLRP1b inflammasome signaling transducers in death mode switching. (a) Sensitivity analysis of the transducer levels. Response of GSDMD activation (upper panel) and caspase-3 activation (down panel) to the fold change from 0.01 to 100 of the standard value for the seven transducers. The *x*-axis and *y*-axis represent the seven transducers and fold change, respectively, while the color shades indicate the concentration variation of GSDMD and caspase-3 activation. (b) Response of pyroptosis induction (upper panel) and apoptosis induction (down panel) to the fold change of the seven transducers. The simulation results are obtained from the model of RAW-asc cells. (c, d) Effects of caspase-1 knockdown on LDH release, caspase-3 activity, and protein activation for indicated times. (e, f) Effects of GSDMD knockdown on LDH release, caspase-3 activity, and protein activation for indicated times.

We next applied our model to predict whether and how each transducer mediate the death mode switch. Sensitivity of the downstream effector proteins, the cleaved GSDMD (N-GSDMD) for pyroptosis, and the cleaved caspase-3 for apoptosis to the change of each transducer is investigated. Expression level of each transducer is individually varied in a range from 0.01-fold to 100-fold of its standard value to inquire the activation of GSDMD and caspase-3 (Figure 3(a)) and the occurrence of pyroptosis and apoptosis (Figure 3(b)). The increase of NLRP1b, caspase-1 (Pro-casp1), or GSDMD (Pro-GSDMD) expression level enhances GSDMD activation, while ASC slightly restrains GSDMD activation (Figure 3(a), upper panel). In contrast, caspase-3 activation is inhibited by the increase of caspase-1 or GSDMD but is promoted by ASC and barely mediated by NLRP1b (Figure 3(a), down panel). However, the cell death behavior suggests that pyroptosis can be regulated by NLRP1b, caspase-1, or GSDMD, while apoptosis only can be regulated by capase-1 and GSDMD (Figure 3(b)). Increase of caspase-1 or GSDMD blocks apoptosis and promotes pyroptosis induction, acting as the death mode switches. Although the increase of ASC restrains GSDMD activation and enhances caspase-3 activation (Figure 3(a)), the corresponding death behavior is barely affected (Figure 3(b)). Simulation results also indicate that variation of caspase-8/9/3 cannot regulate the effector protein activation and thereby the death modes.

To test our prediction, we experimentally knocked down caspase-1 and GSDMD to two different expression levels with short hairpin RNA (shRNA) for caspase-1 and GSDMD, respectively, and detected the activation of the effector proteins, N-GSDMD, and caspase-3 (Figures 3(c)–3(f)). Caspase-1 knockdown limits the LT-induced LDH release. In comparison, caspase-1 knockdown gradually enhances the activation of caspase-3 (Figure 3(c)). Western blotting analysis was additionally performed to study the time responses of the effector proteins under different caspase-1 levels (Figure 3(d)). Decrease of caspase-1 significantly reduces the activation of GSDMD, while gradually enhances the activation of caspase-3, validating our prediction of caspase-1 decrease shown in Figure 3(a). Similar to the results of caspase-1, GSDMD knockdown inhibits LDH release and promotes caspase-3 activation (Figure 3(e)). Time responses of the effector proteins under different GSDMD expression levels are presented in Figure 3(f), supporting our prediction of GSDMD in regulating GSDMD and caspase-3 activation. The prediction of ASC-enhanced caspase-3 activation can be authenticated through comparing with the experimental observations in RAW264.7 cells (Figure S3A-B) that caspase-3 activation cannot occur in RAW, *Casp1*^−/−^ RAW, or *Gsdmd^−/−^* RAW cells but is triggered in *Casp1*^−/−^ RAW-asc or *Gsdmd*^−/−^ RAW-asc cells (Figure 1(e)). Besides, caspase-8 deletion hardly affects LDH release and caspase-3 activation (Figure S3C-D), supporting the nonfunctional role of caspase-8 in death mode switching.

### 2.4. Distinct Cell Death Modes Determined by Caspase-1 and GSDMD Level

**Figure 4:**
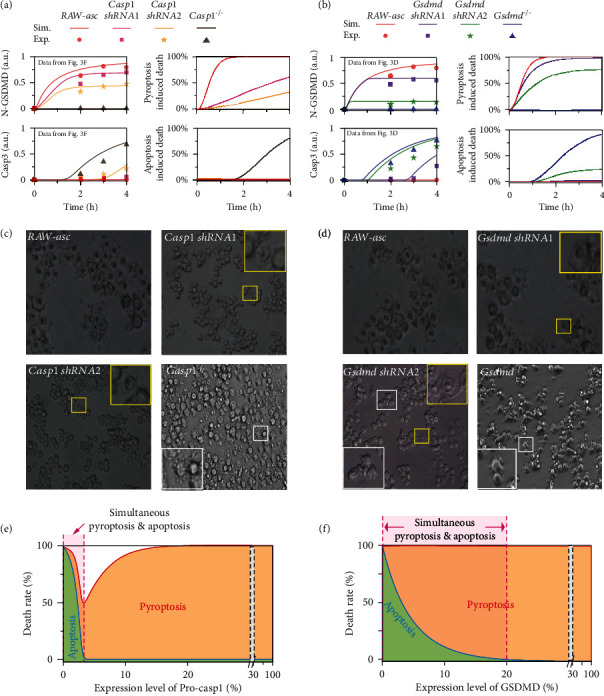
Roles of caspase-1 and GSDMD in determining cell death mode. (a, b) Comparison between model prediction and experimental data of GSDMD/caspase-3 activation, and the prediction of pyroptosis and apoptosis induction under different caspase-1/GSDMD expression levels. (c, d) Cell morphology under different levels of caspase-1/GSDMD. The yellow and white boxes indicate the represented pyroptotic and apoptotic cells under microscopy, respectively. (e, f) The contribution proportions of pyroptosis (brown area) and apoptosis (green area) to total cell death under different caspase-1/GSDMD levels.

Having demonstrated that only caspase-1 and GSDMD can efficiently switch death mode, we next sought to quantify how cell death outcomes are determined by these two transducers. The band intensities in Figures 3(d) and 3(f) are quantified and further compared with model simulations (Figures 4(a) and 4(b), left panels), confirming the high capability of prediction and quantification of our model. Cell death outcomes under the quantified caspase-1 and GSDMD expression levels are predicted as well (Figures 4(a) and 4(b), right panels). Decrease of caspase-1 gradually inhibits pyroptosis. While in *Casp1^−/−^* cells, pyroptosis is completely blocked, and apoptosis occurs alone (Figure 4(a), brown lines in right panel). In contrast, decrease of GSDMD gradually restrains pyroptosis and enhances the induction of apoptosis (Figure 4(b), right panel). The normalized blotting data indicate that caspase-3 activation in *Gsdmd shRNA2* is higher than that in *Casp1 shRNA2* (Figures 4(a) and 4(b), left panels)*. Gsdmd shRNA2* cells are predicted to execute the concurrence of pyroptosis and apoptosis (Figure 4(b), green lines in right panel). Hence, cell death seemingly exhibits different responses to caspase-1 and GSDMD expression level. Microscopy experiment is utilized to validate these predictions. As the cell morphology suggested, only pyroptosis occurs in RAW-asc, *Casp1 shRNA1*, and *Casp1 shRNA2* cells upon LT treatment, while apoptosis can solely be found in *Casp1^−/−^* cells (Figure 4(c)). The experimental observations are quantitatively consistent with our predictions in Figure 4(a) (right panel). Besides, the cell morphology for GSDMD cases (Figure 4(d)) also matches well with our predictions. Only pyroptosis occurs obviously in RAW-asc and *Gsdmd shRNA1* cells. In contrast, both pyroptosis (yellow boxes) and apoptosis (white boxes) can be observed in *Gsdmd shRNA2* cells. Apoptosis is triggered alone in *Gsdmd^−/−^* cells.

To systematically reveal how caspase-1 and GSDMD determine cell death outcomes, we further plot the contribution of pyroptosis and apoptosis to total cell death under different caspase-1/GSDMD expression levels. The contribution diagram suggests that cell death presents a V-shaped biphasic response to caspase-1 decrease (Figure 4(e)). Pyroptosis occurs alone within a wide range of ~3%-100% caspase-1 expression level of RAW-asc cells, and death rate is gradually reduced with caspase-1 decrease within this range. Cooccurrence of pyroptosis and apoptosis appears within an extremely small range and low level of caspase-1 (<~3% of RAW-asc cells). Cell death rate is gradually increased with caspase-1 decrease due to the induction of apoptosis. The extremely small range of caspase-1 for simultaneously inducing pyroptosis and apoptosis is unanimous with the observations that apoptosis is not detected in *Casp1 shRNA1* (~20% caspase-1 level) and *shRNA2* (~10% caspase-1 level) cells (Figure 4(c)). Although decrease of GSDMD switches death modes from pyroptosis to apoptosis as well, the regulation mechanism might be discrepancy. Pyroptosis occurs alone at the range of 20%-100% GSDMD level, whereas simultaneous pyroptosis and apoptosis typically emerge when GSDMD level is lower than 20% (Figure 4(f)). Within the cooccurrence range, the contribution of pyroptosis to cell death becomes weaken, while apoptosis becomes dominant with GSDMD decrease, presenting a death mode switching behavior. This quantitative result well explains the observations that pyroptosis and apoptosis can appear simultaneously in *Gsdmd shRNA2* (~10% GSDMD expression level) but not in *Gsdmd shRNA1* (~40% GSDMD expression level) cells (Figure 4(d)).

### 2.5. Impairment of Caspase-1 and GSDMD Severally Switches Pyroptosis to ASC-Caspase-8-Dependent and Caspase-1-Dependent Apoptosis

**Figure 5:**
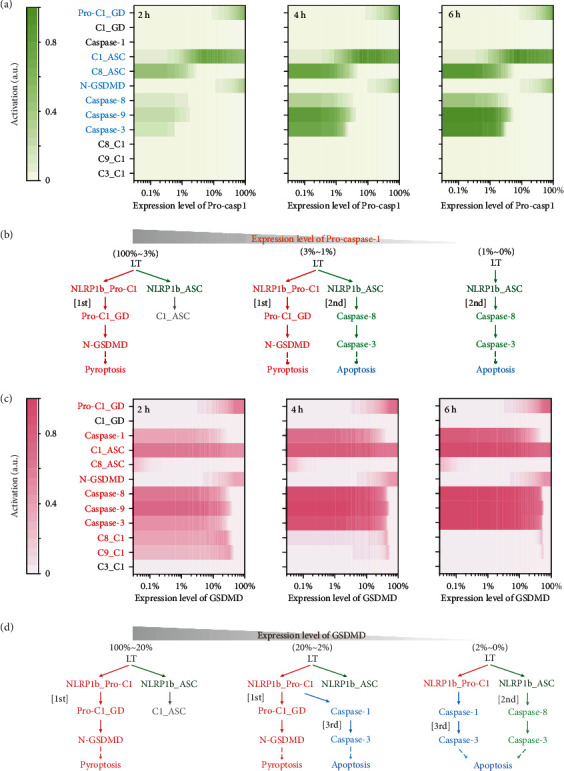
Quantitative analysis of the NLRP1b inflammasome signaling pathways determined by caspase-1 and GSDMD expression levels. (a) Dynamic responses of the key transducers in the three pathways under different caspase-1 levels after 2-, 4-, and 6-hour LT treatment. Each transducer is described by a corresponding variable in Table S2. The binding of one protein to another protein is quantified by measuring the amount of the variables that represent the protein complexes. Amount of Pro-C1_GD (Pro-caspase-1 binding to GSDMD) is represented by the variable pC1_pGD in Tables S2. C1_GD, C1_ASC, C8_ASC, C8_C1, C9_C1, and C3_C1 correspond, respectively, to the variables of C1_pGD, N1b_ASC_pC1, N1b_ASC_pC8, C1_pC8, C1_pC9, and C1_pC3 in Table S2. (b) The proposed scheme of pathway transduction and cell death mode that are quantitatively determined by caspase-1 level. (c) Dynamic responses of the key transducers in the three pathways under different GSDMD levels after 2-, 4-, and 6-hour LT treatment. (d) The proposed scheme of pathway transduction and cell death mode that are quantitatively determined by GSDMD level.

After quantifying the different responses of cell death to caspase-1 and GSDMD, dynamic behavior of transducers and their corresponding formations are examined to dissect the underlying regulation mechanisms. Response of the key transducers and their formations to caspase-1 decrease are analyzed (Figure 5(a)). After 2 hours LT treatment, levels of two transducers, Pro-C1_GD (Pro-caspase-1 binding to GSDMD) and N-GSDMD (cleaved GSDMD), are synchronously reduced with caspase-1 decrease. The range of caspase-1 for these two pyroptotic transducer activation is about 1%-100%. C1_ASC (cleaved caspase-1 binding to ASC) is also gradually declined. Five transducers, C1_GD (cleaved caspase-1 binding to GSDMD), cleaved caspase-1, C8_C1 (caspase-8 binding to caspase-1), C9_C1 (caspase-9 binding to caspase-1), and C3_C1 (caspase-3 binding to caspase-1), remain inactive when varying caspase-1 expression level, while four transducers, C8_ASC (caspase-8 binding to ASC) and cleaved caspase-8/9/3, are synchronously increased with caspase-1 decrease. The minimum threshold of caspase-1 for these apoptotic transducer activation is about 3%. Similar simulation results are obtained at 4 and 6 hours.

Based on the dynamics of these transducers, a quantitative picture of LT-induced NLRP1b inflammasome signaling transduction and cell death outcomes under different caspase-1 expression levels can be drawn (Figure 5(b)). When caspase-1 level is > ~3% of RAW-asc cells, the first/default path of NLRP1b directly recruits Pro-caspase-1 and further cleaves that GSDMD is predominant, solely leading to pyroptosis (Figure 5(b), left panel). Besides, C1_ASC (caspase-1 binding to ASC) keeps at a high level, suggesting NLRP1b-ASC-caspase-1 complex is formed but the downstream is not activated. C8_ASC complex is gradually formed, and apoptotic caspase-8/9/3 are activated without cleaved caspase-1, confirming the second (ASC-caspase-8-dependent) apoptotic path is triggered. Simultaneous pyroptosis and apoptosis induced by the first and second paths occur at an extremely small range of caspase-1 (1%-3%) (Figure 5(b), middle panel), while the second path of apoptosis happens alone when caspase-1 level is <~1% (Figure 5(b), right panel). Caspase-1 remains inactive, and C8_C1, C9_C1, and C3_C1 are unformed, supporting the third path is not triggered with caspase-1 decrease.

Response of the key transducers to GSDMD decrease is quite different from caspase-1 (Figure 5(c)). Upon LT treatment, two transducers, Pro-C1_GD (Pro-caspase-1 binding to GSDMD) and N-GSDMD (cleaved GSDMD), are synchronously reduced with GSDMD decrease. Three transducers, C1_GD (cleaved caspase-1 binding to GSDMD), C1_ASC (cleaved caspase-1 binding to ASC), and C3_C1 (caspase-3 binding to caspase-1), are barely influenced when varying GSDMD level. C1_ASC keeps at a high level, but C1_GD and C3_C1 remain inactive. C8_ASC (caspase-8 binding to ASC) is slightly formed with extremely low level of GSDMD, which is supported by our previous result that ASC oligomerization is somewhat enhanced in *Gsdmd^−/−^* RAW-asc cells [9]. Moreover, six transducers, cleaved caspase-1/8/9/3, C8_C1 (caspase-8 binding to caspase-1), and C9_C1 (caspase-9 binding to caspase-1), are synchronously increased with GSDMD decrease. Hence, a quantitative diagram of inflammasome signaling transduction under different GSDMD levels can be summarized in Figure 5(d). The first path of GSDMD solely leading to pyroptosis is dominant within a broad range of GSDMD level from 20% to 100% (Figure 5(d), left panel). When GSDMD level is within 2%-20%, the first path is attenuated, and pyroptosis still occurs, while caspase-1/8/9/3 are gradually activated, inducing apoptosis through the third path. Thus, simultaneous pyroptosis and apoptosis happen within this range (Figure 5(d), middle panel). GSDMD cannot be cleaved while the caspase-1/8/9/3 are completely activated, leading to apoptosis alone via the third path when GSDMD level is extremely low, i.e., <~2% (Figure 5(d), right panel). C8_ASC (caspase-8 binding to ASC) complex is slightly formed, revealing that the second path of apoptosis can also be partially triggered.

Taken together, both caspase-1 and GSDMD are the “switch node,” which can efficiently trigger the occurrence of solely pyroptosis, apoptosis, or the death mode of simultaneous pyroptosis and apoptosis, whereas their underlying switching mechanisms are different. Decrease of caspase-1 switches Pro-caspase-1-GSDMD dependent pyroptosis to ASC-caspase-8-dependent apoptosis, while GSDMD decrease mainly switches pyroptosis to caspase-1-dependent apoptosis. To trigger the cooccurrence of pyroptosis and apoptosis, the flexible adjustable range of GSDMD (20%-2%) is much broader than caspase-1 (3%-1%), hinting that GSDMD might act as the optimal potential therapeutic target for inducing various death modes.

### 2.6. Cell Death Landscape Unravels Distinct Cooccurrence Modes of Pyroptosis and Apoptosis

**Figure 6:**
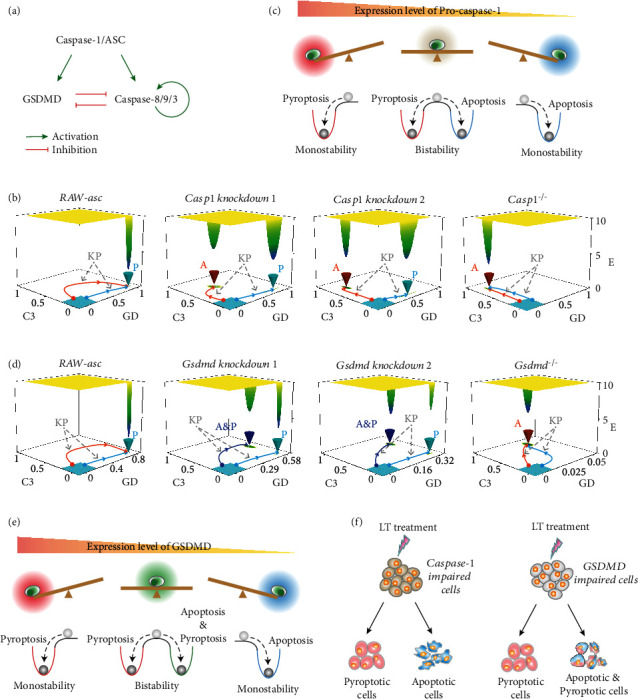
NLRP1b inflammasome-induced cell death landscape topography mediated by caspase-1 and GSDMD levels. (a) Schematic diagram of the coarse-grained pyroptosis-apoptosis circuit model. (b) Cell death landscape topography changes as the reduction of caspase-1 level. A and P, respectively, correspond to the apoptosis well and pyroptosis well. KP represents the kinetic path from a certain initial condition. (c) A diagram of the “seesaw model” that reflects the choice of death modes under different caspase-1 levels. (d) Cell death landscape topography changes as the reduction of GSDMD level. (e) A diagram of the “seesaw model” that reflects the choice of death modes under different GSDMD levels. (f) Schematic representation of the distinct death mode induction in single caspase-1- or GSDMD-impaired cells.

To better understand the mechanisms underlying cell death induction, potential landscape theory that describes the stochastic properties and global stability of the system is employed [33, 37]. It is difficult to use Fokker-Planck equation to solve the evolution probability of the high-dimensional complex system; a coarse-grained pyroptosis-apoptosis circuit model is therefore devised based on the full model. The coarse-grained model is composed of three ordinary differential equations incorporating three modules: the inducer module and the two cell death effector modules (Figure 6(a)). The inducer module is represented by the upstream signaling of caspase-1/ASC, whereas the pyroptosis and apoptosis effector modules are severally indicated by GSDMD and caspase-8/9/3. Both the two effector modules are activated by inducer module. In the apoptosis module, caspase-8/9 cleave caspase-3 and the cleaved caspase-3 also activate caspase-8/9, providing an efficient self-activation of this module for apoptosis induction [29]. The mutual inhibition between the two effector modules may act through their competing for the upstream signaling or directly limiting the activation of each other [18, 28]. Complete description of the coarse-grained model equations and parameters can be found in the Supplementary Data.

Effect of caspase-1 level on the global stability of the system is investigated, and the cell death landscapes on the caspase-3-GSDMD phase space are shown in Figure 6(b). The yellow region corresponds to high potential with low probability of cell death, and the blue region represents low potential with high probability. The RAW-asc cell system exhibits monostable landscape, implying that the system evolves into a unique well from any initial values (Figure 6(b), left panel). The deep well (P well) with low caspase-3 and high GSDMD corresponds to the experimentally observed death mode of pyroptosis (Figure 4(c)). However, when caspase-1 level is largely impaired (*Casp1 knockdown 1*) in cells, the landscape presents two wells. The system eventually evolves into one of the two wells from any initial conditions. Of the two wells, the deep wells with low caspase-3 and high GSDMD or with high caspase-3 and low GSDMD correspond to the death mode of pyroptosis (P well) and apoptosis (A well), respectively. The pyroptosis well is deeper than the apoptosis well, indicating the high occurrence probability of pyroptosis in cells. Decrease of caspase-1 acts as a driving force that reduces pyroptosis well. As caspase-1 level is further impaired (*Casp1 knockdown 2*), the pyroptosis well becomes shallower while the apoptosis well turns deeper, implying that caspase-1 decrease limits pyroptosis but promotes the induction of apoptosis. Deletion of caspase-1 (*Casp1^−/−^*) resulting in the landscape changes from two coexisting death modes to the experimentally observed monostable apoptosis mode (Figure 4(c)), suggesting that only apoptosis occurs from any initial conditions. Caspase-1-mediated cell death landscape topography makes the system behavior like a “seesaw” (Figure 6(c)). The system only falls into the pyroptosis well, and the cells have no chance to undergo apoptosis with high level of caspase-1. However, when caspase-1 is at appropriate low level, the system is located near the balanced region and will selectively fall into the pyroptosis well or apoptosis well, depending on the initial conditions. Extremely low level or deletion of caspase-1 drives the system and exclusively falls into the apoptosis well.

GSDMD level also acts as a driving force that determines the cell death landscape topography. The system changes from monostable pyroptosis well in RAW-asc cells to monostable apoptosis well in *Gsdmd^−/−^* cells (Figure 6(d)), corresponding to the experimentally observed death mode switch from pyroptosis to apoptosis (Figure 4(d)). The landscape topography also presents two wells at appropriate low levels of GSDMD. Of the two wells, the well (P well) with low caspase-3 and high GSDMD corresponds to pyroptosis. Strikingly, the other well with both high caspase-3 and GSDMD corresponds to the induction of simultaneous apoptosis and pyroptosis (A&P well), which is supported by the experimental observation of cooccurrence death mode in GSDMD knockdown cells (Figure 4(d)). The pyroptosis well becomes shallower while the A&P well turns deeper with GSDMD decrease. The GSDMD-mediated landscape topography also presents a seesaw behavior (Figure 6(e)). Different from caspase-1, the system with appropriate low levels of GSDMD will selectively fall into the pyroptosis well or A&P well.

Although simultaneous apoptosis and pyroptosis can be induced by caspase-1 or GSDMD, their landscape topographies imply two potential distinct cooccurrence death modes (Figure 6(f)). In single caspase-1 impaired cells, the mutually antagonistic nature of death modules makes the systems to have an exclusive choice. Each cell will selectively undergo pyroptosis or apoptosis, resulting in the cooccurrence mode at population level. Nonetheless, the fate is distinct in single GSDMD impaired cells. The A&P well suggests that the simultaneous apoptosis and pyroptosis might be even observed in single cells. Apoptosis and pyroptosis could have a “coexisting” state, rather than “mutual inhibition” state in single GSDMD impaired cells. Each cell will selectively undergo pyroptosis or A&P, leading to the cooccurrence mode at population level as well.

## 3. Discussion

Innate immune system forms the first line of host defense against pathogens, and inflammasomes are the core protein complexes. Emerging evidence suggests that inflammasome-induced cell death is highly relative to numerous diseases, including COVID-19 [12, 13]. Pyroptosis incurs diseases due to the release of cytokines, while apoptosis functions as a homeostatic mechanism for disease prevention [11, 19]. Understanding the connectivity of these different cell death types and their potential switching mechanisms are urgently needed. Taken together with previous studies and our experimental observations, we proposed a comprehensive cell death model of the inflammasome signaling. Our study quantitatively elucidates the regulatory mechanisms of the NLRP1b-inflammasome signaling, revealing several new insights into the crosstalk between pyroptosis and apoptosis.

Besides the induction of apoptosis, recent studies demonstrated that the inflammasome-activated caspase-3 can also cleave GSDME to trigger secondary necrosis/pyroptosis [30, 31]. In contrast to the various diseases triggered by pyroptosis, secondary pyroptosis releases fewer inflammatory cytokines, which can reduce the occurrence of severe immune diseases [38]. GSDME was recently reported that can prevent tumor growth through enhancing the cell antitumor function [39]. Exploring the switching mechanisms among the inflammasome-induced pyroptosis, apoptosis and secondary pyroptosis are urgently needed. However, GSDME-induced secondary pyroptosis appears to barely occur in GSDMD- or caspase-1-deficient RAW-asc cells. The release of LDH (Figures 3(c) and 3(e)) and the massively occurrence of apoptosis (Figures 4(c) and 4(d)) in *Gsdmd^−/−^* or *Casp1^−/−^* cells suggest that only a small amount of cells possibly undergo secondary necrosis/pyroptosis. Besides, our previous study indicates that no difference was observed of the LDH release or cell death between *Gsdmd^−/−^* and *Gsdmd^−/−^Gsdme^−/−^* macrophages, confirming that the LDH release and cell death in *Gsdmd^−/−^* or *Casp1^−/−^* cells are not attributed to the GSDME-induced secondary pyroptosis [9]. Actually, a relatively high expression of GSDME is required for overriding apoptotic appearance in caspase-3-activated cells [31], while little GSDME is expressed in RAW 264.7 cells [30]. Thus, to fully address the contribution of secondary pyroptosis in GSDMD- or caspase-1-deficient macrophages, further analysis using the cells with high expression level of GSDME, such as bone marrow-derived macrophages (BMDM), is required.

Targeting pyroptosis or apoptosis induced by inflammasomes is suggested to be a promising strategy in COVID-19 and other disease treatment [1, 13, 20, 40]. Focusing on exploring the efficient control strategy of the transducers is therefore a significant issue [41]. Our results clearly demonstrate that only the decrease of caspase-1 and GSDMD levels can individually switch death modes from pyroptosis to apoptosis (Figure 3(b)). Both caspase-1 and GSDMD are the attractive targets. Actually, several caspase-1/GSDMD inhibitor drugs have recently been developed for disease treatment. The structure-based discovery of CZL80 offers much therapeutic potential for febrile seizures and later enhanced epileptogenic susceptibility through inhibiting caspase-1 [42]. 2-4-Diaminopyrimidine, an important fragment in the inhibition of human caspase-1, is designed to be applied for the treatment of Alzheimer's disease [43]. The FDA-approved drug disulfiram (DSF) that was recently found to inhibit pyroptosis through blocking GSDMD pore formation is effective in a large number of inflammatory diseases [44]. Necrosulfonamide (NSA) has been previously demonstrated that can directly inhibit the pore formation of GSDMD, blocking pyroptosis and interleukin-1*β* release without inhibiting other innate immune pathways [45]. However, a subsequent study suggests that NSA can also inhibit inflammasome upstream of GSDMD, blocking pyroptosis independent of GSDMD targeting [46]. Further research is therefore urgently required to clearly address the different mechanisms of NSA for inflammatory disease treatment. For COVID-19 treatment, the caspase-1 inhibitor, belnacasan (VX765), is recently considered to have the therapeutic potential [47, 48]. Besides, DSF and dimethyl fumarate (DMF) have recently been described to inhibit GSDMD and confer beneficial effects in treating COVID-19 [44, 49]. An observational study reveals a significantly reduced risk of SARS-CoV-2 infection with DSF treatment for alcoholism [44], while SARS-CoV-2 infection is self-limiting without any specific treatment in multiple sclerosis patients treated with DMF [49]. Our results also suggest that varying the expression level of NLRP1b, ASC, or caspase-3/8/9 could not efficiently switch death mode (Figure 3(b)). Targeting on these transducers in the inflammasome signaling might be invalid for disease prevention and treatment.

Another important issue is what is the best level of inhibitors to intervene. Pyroptosis is indispensable mechanism for cells to implement immune responses. Moderate pyroptosis is helpful to cell homeostasis, effectively protecting the host and resisting infection and endogenous risk factors [50]. However, excessive pyroptosis incurs cytokine storm and various diseases [1, 10, 11]. Appropriate apoptosis also facilitates the clearance of infected cells, playing essential roles for disease inhibition [10, 19]. Our quantitative analysis shows that the contribution of pyroptosis to total cell death is decreased, while apoptosis becomes important with the reduction of caspase-1 or GSDMD (Figures 4(e) and 4(f)). Pyroptosis trends to switch to apoptosis with an extremely low threshold of caspase-1 level, but with a relatively high threshold of GSDMD. A cooccurrence of moderate pyroptosis and apoptosis might provide a promising and powerful therapeutic strategy for inflammatory diseases, including COVID-19. Nonetheless, the cooccurrence death mode occurs within an extremely low level range of caspase-1 but within a much broader range of GSDMD, giving a flexible requirement of caspase-1 inhibitor for disease treatment. Moreover, caspase-1-impaired cells employ the ASC-caspase-8-dependent pathway to switch death modes from pyroptosis to apoptosis. However, besides the ASC-caspase-8-dependent pathway, GSDMD-impaired cells can also utilize the caspase-1-dependent pathway. Of the two switch strategies, ASC is not required for GSDMD. Overall, the cooccurrence ranges and pathway choices support that GSDMD should be considered as the optimal potential therapeutic target of the NLRP1b inflammasome signaling.

Through providing a more physical description of the stochastic dynamic and global stability of the biological systems, recently developed potential landscape theory is a powerful approach for identifying new functional states or unknown regulatory mechanisms [33, 37]. Most recently, a new cell-aging fate induced by overexpression of the lysine deacetylase Sir2 was found by using this approach [51]. Besides, an unexpected observation of the lineage specifiers that are considered as pluripotency rivals can facilitate reprogramming and replace reprogramming factors of a corresponding lineage-specifying potential, which was successfully clarified with landscape analysis [52]. In this study, we presented the first landscape of cell death induced by inflammasome signaling. Unexpectedly, two distinct cooccurrence death modes of pyroptosis and apoptosis are found by analyzing the landscape topography. A “mutual inhibition” relationship between pyroptosis and apoptosis pathways is generally assumed as they can completely block the activation of each other [9, 18, 28]. The landscape topography of caspase-1 impaired system with a pyroptosis well and an apoptosis well supports the “mutual inhibition” relationship. Each cell selectively undergoes pyroptosis or apoptosis, leading the cooccurrence at population level. However, a new well, A&P well, is found by analyzing the landscape topography of GSDMD impaired system, suggesting that simultaneous apoptosis and pyroptosis might be even observed in single cells. Thus, pyroptosis and apoptosis in single cells could be both “coexisting” or “mutual inhibition,” depending on the initial conditions. Actually, our recent study found that simultaneous apoptosis and necroptosis can occur in single cells as well [53], revealing that the “coexisting” state of different cell death types might be a fundamental property of cells. Thus, there should be a “speed competition” between pyroptosis and apoptosis pathways in such single cells. Cell fate should be determined by the pathway that first reaches the destination. The “seesaw model” well describes how the three death modes, i.e., pyroptosis, apoptosis, and A&P, are controlled by caspase-1 or GSDMD in single cells. Compared with the exclusive pyroptosis or apoptosis, A&P cells can provide a new therapeutic option for diseases.

Besides NLRP1b, the similar switching behaviors are also observed in many other inflammasome signaling. GSDMD or caspase-1 deletion switches NLRP3 inflammasome-mediated pyroptosis to apoptosis was previously reported in both RAW-asc cells and BMDM [3]. Besides, our recent study observed the same effect of GSDMD or caspase-1 deletion on cell death induced by AIM2 inflammasome in J774A.1 cells [9]. Caspase-1 deletion in NLRP3 or AIM2 inflammasomes was confirmed that triggers the ASC-caspase-8-dependent apoptosis in BMDM [54, 55], while GSDMD deletion in these inflammasomes induces caspase-1-dependent apoptosis [18]. The NLRC4 inflammasome-induced ASC-caspase-8-dependent apoptosis in caspase-1-deficient cells and caspase-1-dependent apoptosis in GSDMD-deficient cells were also demonstrated in peritoneal macrophages [9] and BMDM [18]. Overall, these observations imply that the switching strategy in caspase-1- or GSDMD-impaired cells should be a general property upon the activation of different inflammasomes. Notably, NLRP3- or AIM2-induced caspase-1 activation and pyroptosis require ASC, while NLRC4 or NLRP1b can induce pyroptosis independent of ASC [56]. The default pyroptosis pathway is ASC-caspase-1-GSDMD in NLRP3 or AIM2 inflammasome signaling, while it is Pro-caspase-1-GSDMD in NLRC4 or NLRP1b. Although the apoptosis pathways are similar in caspase-1/GSDMD-impaired cells upon the activation of different inflammasomes, their default pyroptosis pathways are different. Overall, we hope these new insights provided by this study can offer the guidance of potential strategies and drug development for disease treatment, especially COVID-19.

## 4. Materials and Methods

### 4.1. Cell Cultures and Stimulation

RAW 264.7 and J774A.1 cells were obtained from ATCC. RAW-asc was constructed on RAW 264.7 as previously described [9]. All cells were grown in Dulbecco's modified Eagle's medium (DMEM) supplemented with 10% fetal bovine serum (FBS) (10099-141, Gibco), penicillin (100 units/ml), and streptomycin (100 *μ*g/ml). For NLRP1b inflammasome activation, the cells (RAW 264.7, RAW-asc, or J774A.1) were cultured with LF (2 *μ*g/ml) (List Biological Labs, #172C) together with PA (2 *μ*g/ml) (List Biological Labs, #171E) for the indicated times.

### 4.2. Generation of Knockout and Knockdown Cell Lines

The targeting sequence in the gRNA vector was 5′-TCTCTAAAAAAGGGCCCC-3′ for mouse *caspase-1*; 5′-TGCAACAGCTTCGGAGTCG-3′ for mouse *Gsdmd*. The plasmids (vector pBOB) harboring the gene gRNA sequences and Cas9 gene were transfected into 293T in the presence of lentivirus helper plasmids, and the supernatants were collected after 24 h. The viruses were then used to infect RAW-asc cells and J774A.1 cells. Knockouts were confirmed by immunoblots and further confirmed by sequencing. For genetic knockdown, the 293T cells were transfected with lentivirus helper plasmids and plasmids of short hairpin RNA (shRNA) for shGSDMD (sense sequence: TGGTGCTTGACTCTGGAGA) or shCaspase-1 (sense sequence: GATTTCTTAACGGATGCAA), and the supernatants were collected after 48 h. The viruses were then used to infect RAW-asc cells. Knockdown of GSDMD and caspase-1 was confirmed by immunoblots.

### 4.3. Immunoblot Analysis

Cell lysates together with culture supernatants were collected by adding 5× sample buffer (50% glycerol, 10% SDS, 5% 2-mercaptoethanol, 0.02% bromophenol blue, and 250 mM pH 6.8 Tris-HCl) for immunoblot analysis. Proteins were separated by 10%–15% polyacrylamide gels, followed by electrophoretic transfer to PVDF membranes (IPVH00010, Millipore). The membrane was then blocked by incubation with 5% BSA before being incubated with primary antibodies. Antibodies used include caspase-1 (clone 4B4), a kind gift from Vishva M. Dixit (Genentech, USA), caspase-3 (9662, Cell Signaling), pro-caspase-8 (4790, Cell Signaling), cleaved-caspase-8 (9429, Cell Signaling), caspase-9 (9508, Cell Signaling), GSDMD (ab209845, Abcam), ASC (67824, Cell Signaling), and GAPDH (AC002, ABclonal).

### 4.4. LDH and Cell Viability Assay

Cell cytotoxicity was determined by using Cytotoxicity LDH Assay Kit-WST (CK12-500-wells, Dojindo). The number of viable cells was determined by using the CellTiter-Glo Luminescent Cell Viability Assay Kit according to the manufacturer's instructions (G7571, Promega).

### 4.5. Microscopy Imaging of Cell Death

To examine cell death morphology, cells were treated as indicated in 12-well plates or 35 mm glass bottom dishes for image capture. Static bright-field images of cells were captured using Zeiss LSM 780 at room temperature. The pictures were processed using ImageJ or the ZEN 2012 Image program.

### 4.6. Measurement of Caspase-1, Caspase-3/7, and Caspase-8 Activities

Caspase-1, caspase-3/7, and caspase-8 activities were determined by using a caspase-Glo 1 (Promega, G9951), caspase-Glo 3/7 (G8092, Promega), or caspase-8 assay kit (G8202, Promega) according to the manufacturer's instructions. Cells were seeded in 96-well plate with white wall (Nunc). After treatment, an equal volume of caspase-Glo 1, caspase-Glo 3/7, or caspase-8 reagent was added to the cell culture medium and shaken for 30 min. Luminescent recording was performed with POLARstar Omega (BMG Labtech).

### 4.7. Model Construction of NLRP1b Inflammasome Signaling

Ordinary differential equation- (ODE-) based modeling is a well-established approach and has been widely used to quantitatively study the cellular regulatory mechanism [33, 53, 57]. The cell state can be described by the component concentrations (*C*_1_, *C*_2_, ⋯). Based on the law of mass action, the reaction rates are dependent on these concentrations and the kinetic parameters (*k*_1_, *k*_2_, ⋯). The model is formulated as a set of coupled ODEs to describe the time evolution of component concentrations in terms of the following general equation:

(1) $$\begin{array}{c} \frac{{dC}_{i}}{dt}=\overset{n}{\underset{j=1}{\sum }}v_{ij}\cdot q_{j}\cdot ,\left(i=1,\cdots ,m\right), \end{array}$$

where *dC*_*i*_/*dt* is the concentration changing rate of component *i* with time. *m* represents the number of components with the concentration *C*_*i*_. *n* is the number of reactions with the rate *q*_*j*_, and *v*_*ij*_ denotes the element of stoichiometric matrix that links the reaction rates of *qi* with component *C*_*i*_. Complete description of model reactions and ODEs is given in Tables S1 and S2. The ODE model is developed and simulated with MATLAB, and the ODE 15 s function of MATLAB is used to solve ODEs. The zipped source code file can be found in https://github.com/jianweishuai/NLRP1b-inflammasome.

### 4.8. Parameter Values and Initial Amount Selection

All parameters in the full NLRP1b signaling model are firstly restricted to be within the typical biological ranges according to the reaction type. Then, we further estimated the parameters based on the experimental data or earlier literature [9, 32, 58]. The parameters are mostly determined by a global optimization method that minimizes the deviation between simulation results and western blotting data. The deviation is characterized by using the correlation coefficient, *R*-square, which is determined as the following functions:

(2) $$\begin{array}{c} R^{2}=1-\frac{\sum_{i=1}^{n}{\left(y_{\exp}\left(t_{i}\right)-y_{\text{sim}}\left(t_{i}\right)\right)}^{2}}{\sum_{i=1}^{n}{\left(y_{\exp}\left(t_{i}\right)-\bar{y_{\exp}}\right)}^{2}}, \end{array}$$

where *y*_exp_(*t*_*i*_) and *y*_sim_(*t*_*i*_) are the experimental data and simulated data at time *t*_*i*_, respectively. ${\bar{\text{y}}}_{\exp}$ is the average value of the quantified western blotting data. The parameters that are not available from experimental data are derived from literature or estimated within a biologically plausible range [32]. All the parameter descriptions and values are presented in Table S3. Initial amounts of all the components are listed in Table S2, which are obtained from earlier literature or estimation [34].

### 4.9. Potential Landscape Computation

The stochastic dynamics of the inflammasome signaling system can be described by Langevin equation, i.e., *dC*_*i*_(*t*)/*dt* = *F*(*C*) + *η*(*t*), where *C* represents the concentration of the molecules or gene expression levels. *F*(*C*) represents the driving force that describing the dynamics of the system. The noise term *η*(*t*) adopts the independent additive white Gaussian noise, 〈*η*(*t*)〉 = 0 and 〈*η*(*t*)*η*(*t*′)〉 = 2*Dδ*(*t* − *t*′). *δ*(*t*) is the Dirac delta function, and *D* is the level of noise magnitude. The probability evolution *P* for the system can be reflected by the Fokker-Planck equation:

(3) $$\begin{array}{c} \frac{\partial P\left(C,t\right)}{\partial t}=-\sum \frac{\partial }{\partial C_{i}}\left[F_{i}\left(C\right)P\left(C,\text{t}\right)\right]+\sum D_{i}\frac{\partial^{2}}{\partial {C_{i}}^{2}}P\left(C,\text{t}\right). \end{array}$$

Thus, the dimensionless potential *E* and steady state probability distribution *P*_*ss*_ of the system are given by the Boltzmann relation; that is, *E* = −ln(*P*_*ss*_). Since it is still hard to directly solve the diffusion equation of high-dimensional system, a coarse-grained model comprising three modules is proposed. Detailed description of the three-dimensional model and the potential landscape analysis can be found in the Supplementary Materials.

## Data Availability

The zipped source code file can be found in https://github.com/jianweishuai/NLRP1b-inflammasome.

## Supplementary Materials

Supplementary Materials The detailed description of the coarse-grained pyroptosis-apoptosis circuit model is presented in the Supplementary Text in the Supplementary Materials. Figure S1: analysis of Casp1 or Gsdmd knockout in J774A.1 cells. Figure S2: kinetic scheme of the NLRP1b inflammasome-induced cell death signaling model. Figure S3: analysis of Casp1, Gsdmd, or Casp8 knockout in regulating cell death. Table S1: reactions and reaction rates. Table S2: ordinary differential equations and initial amounts. Table S3: parameters of the model.

## References

[1] Lamkanfi M., Dixit V. M. (2014). Mechanisms and functions of inflammasomes. *Cell*.

[2] Schroder K., Tschopp J. (2010). The inflammasomes. *Cell*.

[3] He W., Wan H., Hu L. (2015). Gasdermin D is an executor of pyroptosis and required for interleukin-1*β* secretion. *Cell Research*.

[4] Shi J., Zhao Y., Wang K. (2015). Cleavage of GSDMD by inflammatory caspases determines pyroptotic cell death. *Nature*.

[5] Kayagaki N., Stowe I. B., Lee B. L. (2015). Caspase-11 cleaves gasdermin D for non-canonical inflammasome signalling. *Nature*.

[6] Guo H., Callaway J. B., Ting J. P. Y. (2015). Inflammasomes: mechanism of action, role in disease, and therapeutics. *Nature Medicine*.

[7] Chen X., He W., Hu L. (2016). Pyroptosis is driven by non-selective gasdermin-D pore and its morphology is different from MLKL channel-mediated necroptosis. *Cell Research*.

[8] Ding J., Wang K., Liu W. (2016). Pore-forming activity and structural autoinhibition of the gasdermin family. *Nature*.

[9] Zhang P., Liu Y., Hu L. (2021). NLRC4 inflammasome-dependent cell death occurs by a complementary series of three death pathways and determines lethality in mice. *Science Advances*.

[10] Liu X., Xia S., Zhang Z., Wu H., Lieberman J. (2021). Channelling inflammation: gasdermins in physiology and disease. *Nature Reviews Drug Discovery*.

[11] Orning P., Lien E., Fitzgerald K. A. (2019). Gasdermins and their role in immunity and inflammation. *Journal of Experimental Medicine*.

[12] Vora S. M., Lieberman J., Wu H. (2021). Inflammasome activation at the crux of severe COVID-19. *Nature Reviews Immunology*.

[13] Karki R., Sharma B. R., Tuladhar S. (2021). Synergism of TNF-*α* and IFN-*γ* triggers inflammatory cell death, tissue damage, and mortality in SARS-CoV-2 infection and cytokine shock syndromes. *Cell*.

[14] Lamers M. M., Haagmans B. L. (2022). SARS-CoV-2 pathogenesis. *Nature Reviews Microbiology*.

[15] Huang C., Wang Y., Li X. (2020). Clinical features of patients infected with 2019 novel coronavirus in Wuhan, China. *The Lancet*.

[16] Qiu T., Liang S., Dabbous M., Wang Y., Han R., Toumi M. (2020). Chinese guidelines related to novel coronavirus pneumonia. *Journal of Market Access & Health Policy*.

[17] Huet T., Beaussier H., Voisin O. (2020). Anakinra for severe forms of COVID-19: a cohort study. *The Lancet Rheumatology*.

[18] Tsuchiya K., Nakajima S., Hosojima S. (2019). Caspase-1 initiates apoptosis in the absence of gasdermin D. *Nature Communications*.

[19] Rathmell J. C., Thompson C. B. (2002). Pathways of apoptosis in lymphocyte development, homeostasis, and disease. *Cell*.

[20] Liu Y., Garron T. M., Chang Q. (2021). Cell-type apoptosis in lung during SARS-CoV-2 infection. *Pathogens*.

[21] Ren Y., Shu T., Wu D. (2020). The ORF3a protein of SARS-CoV-2 induces apoptosis in cells. *Cellular & Molecular Immunology*.

[22] Van Opdenbosch N., Gurung P., Vande Walle L., Fossoul A., Kanneganti T.-D., Lamkanfi M. (2014). Activation of the NLRP1b inflammasome independently of ASC-mediated caspase-1 autoproteolysis and speck formation. *Nature Communications*.

[23] von Moltke J., Trinidad N. J., Moayeri M. (2012). Rapid induction of inflammatory lipid mediators by the inflammasome in vivo. *Nature*.

[24] Boyden E. D., Dietrich W. F. (2006). Nalp1b controls mouse macrophage susceptibility to anthrax lethal toxin. *Nature Genetics*.

[25] Mathur A., Hayward J. A., Man S. M. (2018). Molecular mechanisms of inflammasome signaling. *Journal of Leukocyte Biology*.

[26] Van Opdenbosch N., Van Gorp H., Verdonckt M. (2017). Caspase-1 engagement and TLR-induced c-FLIP expression suppress ASC/caspase-8-dependent apoptosis by inflammasome sensors NLRP1b and NLRC4. *Cell Reports*.

[27] Heilig R., Dilucca M., Boucher D. (2020). Caspase-1 cleaves Bid to release mitochondrial SMAC and drive secondary necrosis in the absence of GSDMD. *Life Science Alliance*.

[28] Taabazuing C. Y., Okondo M. C., Bachovchin D. A. (2017). Pyroptosis and apoptosis pathways engage in bidirectional crosstalk in monocytes and macrophages. *Cell Chemical Biology*.

[29] McComb S., Chan P. K., Guinot A. (2019). Efficient apoptosis requires feedback amplification of upstream apoptotic signals by effector caspase-3 or -7. *Science Advance*.

[30] Rogers C., Fernandes-Alnemri T., Mayes L., Alnemri D., Cingolani G., Alnemri E. S. (2017). Cleavage of DFNA5 by caspase-3 during apoptosis mediates progression to secondary necrotic/pyroptotic cell death. *Nature Communications*.

[31] Wang Y., Gao W., Shi X. (2017). Chemotherapy drugs induce pyroptosis through caspase-3 cleavage of a gasdermin. *Nature*.

[32] Albeck J. G., Burke J. M., Spencer S. L., Lauffenburger D. A., Sorger P. K. (2008). Modeling a snap-action, variable-delay switch controlling extrinsic cell death. *PLoS Biology*.

[33] Li X., Jin J., Zhang X. (2021). Quantifying the optimal strategy of population control of quorum sensing network in _Escherichia coli_. *Npj Systems Biology and Applications*.

[34] Schwanhaeusser B., Busse D., Li N. (2011). Global quantification of mammalian gene expression control. *Nature*.

[35] Dick M. S., Sborgi L., Ruhl S., Hiller S., Broz P. (2016). ASC filament formation serves as a signal amplification mechanism for inflammasomes. *Nature Communications*.

[36] Vajjhala P. R., Lu A., Brown D. L. (2015). The inflammasome adaptor ASC induces procaspase-8 death effector domain filaments. *Journal of Biological Chemistry*.

[37] Li C., Wang J. (2014). Landscape and flux reveal a new global view and physical quantification of mammalian cell cycle. *Proceedings of the National Academy of Sciences of the United States of America*.

[38] Aizawa E., Karasawa T., Watanabe S. (2020). GSDME-dependent incomplete pyroptosis permits selective IL-1*α* release under caspase-1 inhibition. *iScience*.

[39] Zhang Z. B., Zhang Y., Xia S. Y. (2020). Gasdermin E suppresses tumour growth by activating anti-tumour immunity. *Nature*.

[40] Alpan O., Gupta R., Latterich M., Hubka M., Bukhari Z., Ndhlovu L. (2021). D020 role of cellular caspases and therapeutic potential of a pan-caspase inhibitor, emricasan, in COVID-19. *Annals of Allergy Asthma & Immunology*.

[41] Yang Q., Jian X., Syed A. A. S. (2022). Structural comparison and drug screening of spike proteins of ten SARS-CoV-2 variants. *Research*.

[42] Tang Y., Feng B., Wang Y. (2020). Structure-based discovery of CZL80, a caspase-1 inhibitor with therapeutic potential for febrile seizures and later enhanced epileptogenic susceptibility. *British Journal of Pharmacology*.

[43] Kumi R. O., Soremekun O. S., Issahaku A. R., Agoni C., Olotu F. A., Soliman M. E. S. (2020). Exploring the ring potential of 2,4-diaminopyrimidine derivatives towards the identification of novel caspase-1 inhibitors in Alzheimer’s disease therapy. *Journal of Molecular Modeling*.

[44] Hu J. J., Liu X., Xia S. (2020). FDA-approved disulfiram inhibits pyroptosis by blocking gasdermin D pore formation. *Nature Immunology*.

[45] Rathkey J. K., Zhao J. J., Liu Z. H. (2018). Chemical disruption of the pyroptotic pore-forming protein gasdermin D inhibits inflammatory cell death and sepsis. *Science Immunology*.

[46] Rashidi M., Simpson D. S., Hempel A. (2019). The pyroptotic cell death effector gasdermin D is activated by gout-associated uric acid crystals but is dispensable for cell death and IL-1*β* release. *Journal of Immunology*.

[47] Li S., Huang H., Wei Q. (2021). Depression of pyroptosis by inhibiting caspase-1 activation improves neurological outcomes of kernicterus model rats. *ACS Chemical Neuroscience*.

[48] Flores J., Noel A., Foveau B., Lynham J., Lecrux C., LeBlanc A. C. (2018). Caspase-1 inhibition alleviates cognitive impairment and neuropathology in an Alzheimer's disease mouse model. *Nature Communications*.

[49] Humphries F., Shmuel-Galia L., Ketelut-Carneiro N. (2020). Succination inactivates gasdermin D and blocks pyroptosis. *Science*.

[50] Li Q., Wang Q., Guan H., Zhou Y., Liu L. (2021). Schisandrin inhibits NLRP1 inflammasome-mediated neuronal pyroptosis in mouse models of Alzheimer’s disease. *Neuropsychiatric Disease and Treatment*.

[51] Li Y., Jiang Y., Paxman J. (2020). A programmable fate decision landscape underlies single-cell aging in yeast. *Science*.

[52] Shu J., Wu C., Wu Y. (2013). Induction of pluripotency in mouse somatic cells with lineage specifiers. *Cell*.

[53] Li X., Zhong C., Wu R. (2021). RIP1-dependent linear and nonlinear recruitments of caspase-8 and RIP3 respectively to necrosome specify distinct cell death outcomes. *Protein & Cell*.

[54] Sagulenko V., Thygesen S. J., Sester D. P. (2013). AIM2 and NLRP3 inflammasomes activate both apoptotic and pyroptotic death pathways via ASC. *Cell Death & Differentiation*.

[55] Pierini R., Juruj C., Perret M. (2012). AIM2/ASC triggers caspase-8-dependent apoptosis in Francisella-infected caspase-1-deficient macrophages. *Cell Death & Differentiation*.

[56] Malik A., Kanneganti T. D. (2017). Inflammasome activation and assembly at a glance. *Journal of Cell Science*.

[57] Qi H., Li X., Jin Z., Simmen T., Shuai J. (2020). The oscillation amplitude, not the frequency of cytosolic calcium, regulates apoptosis induction. *iScience*.

[58] Anderson M. W., Moss J. J., Szalai R., Lane J. D. (2019). Mathematical modeling highlights the complex role of AKT in TRAIL-induced apoptosis of colorectal carcinoma cells. *iScience*.

