# Supplementary material for: Caspase-1 and Gasdermin D Afford the Optimal Targets with Distinct Switching Strategies in NLRP1b Inflammasome-Induced Cell Death
Source: Research (Wash D C). 2022 Jul 19;2022:9838341. doi: 10.34133/2022/9838341 (PMC9343085; doi:10.34133/2022/9838341)
Supplement: Supplementary Materials — The detailed description of the coarse-grained pyroptosis-apoptosis circuit model is presented in the Supplementary Text in the Supplementary Materials. Figure S1: analysis of Casp1 or Gsdmd knockout in J774A.1 cells. Figure S2: kinetic scheme of the NLRP1b inflammasome-induced cell death signaling model. Figure S3: analysis of Casp1, Gsdmd, or Casp8 knockout in regulating cell death. Table S1: reactions and reaction rates. Table S2: ordinary differential equations and initial amounts. Table S3: parameters of the model. [file 9838341.f1.doc]

Supplementary Materials

**Supplementary Text**

***Deterministic coarse-grained model***

Deterministic dynamics of the coarse-grained pyroptosis-apoptosis circuit model can be described by the following three dimension ordinary differential equations:

$\begin{matrix} \frac{d[C1]}{dt}=a_{1}-d_{1}[C1]+k_{1}[LF]\frac{(1-[C1])^{n_{1}}}{(1-[C1])^{n_{1}}+j_{1}^{n_{1}}}+k_{2}[GD]\frac{(1-[C1])^{n_{2}}}{(1-[C1])^{n_{2}}+j_{2}^{n_{2}}}, \\ \frac{d[GD]}{dt}=a_{2}-d_{2}[GD]+k_{3}[C1]\frac{(1-[GD])^{n_{3}}}{(1-[GD])^{n_{3}}+j_{3}^{n_{3}}}-k_{4}[C3]\frac{(1-[GD])^{n_{4}}}{(1-[GD])^{n_{4}}+j_{4}^{n_{4}}}, \\ \frac{d[C3]}{dt}=a_{3}-d_{3}[C3]+k_{5}[C1]\frac{(1-[C3])^{n_{5}}}{(1-[C3])^{n_{5}}+j_{5}^{n_{5}}}+k_{6}[C3]\frac{(1-[C3])^{n_{6}}}{(1-[C3])^{n_{6}}+j_{6}^{n_{6}}}-k_{7}[GD]\frac{[C3]^{n_{7}}}{[C3]^{n_{7}}+j_{7}^{n_{7}}}. \end{matrix}$where *a*_1_ = 0.014, *a*_2_ = 0.027, *a*_3_ = 0.013, *d*_1_ = 0.027, *d*_2_ = 0.094, *d*_3_ = 0.066, *k*_1_ = 0.16, *k*_2_ = 0, *k*_3_ = 0.47, *k*_4_ = 3.2, *k*_5_ = 9.0, *k*_6_ = 1.3, *k*_7_ = 0.56, *j*_1_ = 0.005, *j*_2_ = 49.0, *j*_3_ = 0.1, *j*_4_ = 12.0, *j*_5_ = 39.0, *j*_6_ = 0.6, *j*_7_ = 0.1, *n*_1_ = 2.0, *n*_2_ = 3.0, *n*_3_ = 2.0, *n*_4_ = 1.0, *n*_5_ = 3.0, *n*_6_ = 2.0, *n*_7_ = 1.0 and *LF* = 0.7.

Bifurcation diagrams that reflect the stability of the system in response to caspase-1 and GSDMD variation are shown below:


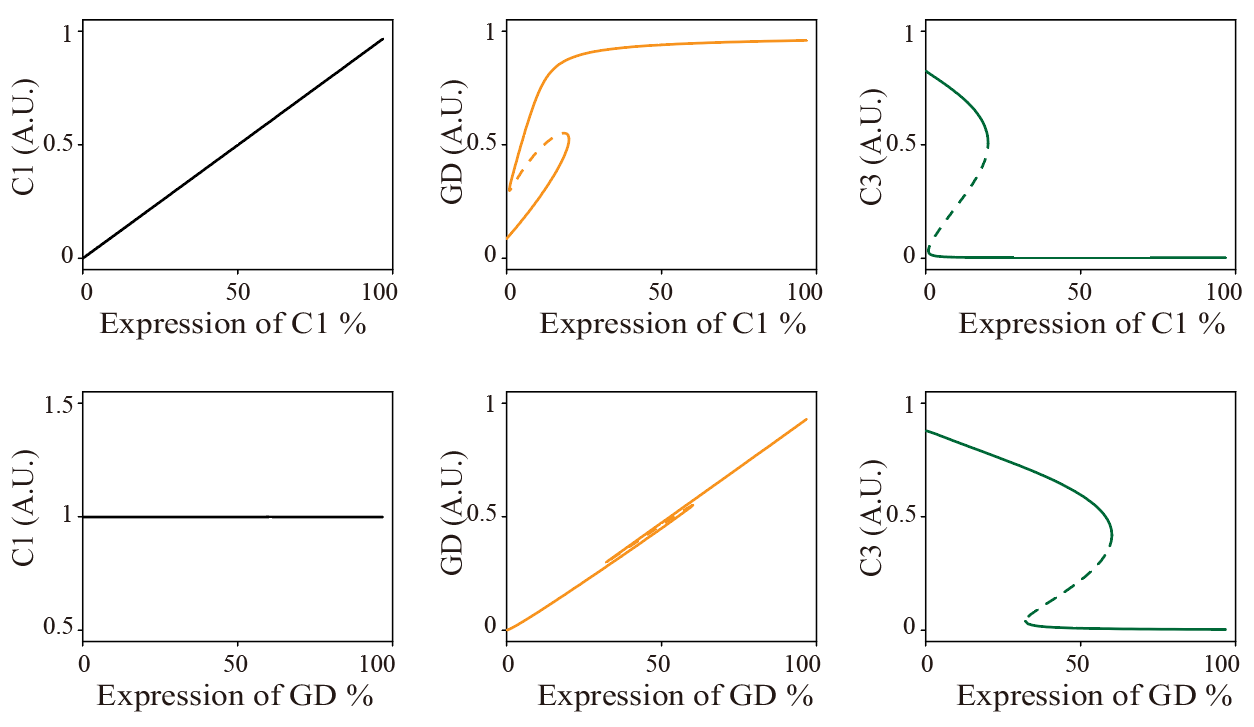


***Stochastic coarse-grained model***

Stochastic dynamics of the coarse-grained pyroptosis-apoptosis circuit model can be described by the following Langevin equations in which three noise terms, *ξ*_C1_, *ξ*_GD_ and *ξ*_C3_, are added to the deterministic equations:

$\begin{matrix} \frac{d[C1]}{dt}=a_{1}-d_{1}[C1]+k_{1}[LF]\frac{(1-[C1])^{n_{1}}}{(1-[C1])^{n_{1}}+j_{1}^{n_{1}}}+k_{2}[GD]\frac{(1-[C1])^{n_{2}}}{(1-[C1])^{n_{2}}+j_{2}^{n_{2}}}+\xi_{C1}, \\ \frac{d[GD]}{dt}=a_{2}-d_{2}[GD]+k_{3}[C1]\frac{(1-[GD])^{n_{3}}}{(1-[GD])^{n_{3}}+j_{3}^{n_{3}}}-k_{4}[C3]\frac{(1-[GD])^{n_{4}}}{(1-[GD])^{n_{4}}+j_{4}^{n_{4}}}+\xi_{GD}, \\ \frac{d[C3]}{dt}=a_{3}-d_{3}[C3]+k_{5}[C1]\frac{(1-[C3])^{n_{5}}}{(1-[C3])^{n_{5}}+j_{5}^{n_{5}}}+k_{6}[C3]\frac{(1-[C3])^{n_{6}}}{(1-[C3])^{n_{6}}+j_{6}^{n_{6}}}-k_{7}[GD]\frac{[C3]^{n_{7}}}{[C3]^{n_{7}}+j_{7}^{n_{7}}}+\xi_{C3}. \end{matrix}$The noise terms are modeled as three independent white Gaussian processes with magnitudes *D*_C1_, *D*_GD_ and *D*_C3_,

$$\left\langle\xi_{i}(t)\xi_{j}(t')\right\rangle=\sqrt{D_{i}D_{j}}\delta_{ij}\delta(t-t') and i,j\in\{C1,GD,C3\}.$$

The associated three-dimensional Fokker-Planck equation for the dynamics of the probability distribution 𝑝

$$\frac{\partial p}{\partial t}=-\frac{\partial}{\partial[C1]}(F_{C1}p)-\frac{\partial}{\partial[GD]}(F_{GD}p)-\frac{\partial}{\partial[C3]}(F_{C3}p)+D_{C1}\frac{\partial^{2}p}{\partial[C1]^{2}}+D_{GD}\frac{\partial^{2}p}{\partial[GD]^{2}}+D_{C3}\frac{\partial^{2}p}{\partial[C3]^{2}}.$$

The formula *E=*−*ln*(*p*) to obtain the potential landscape, and the initial value is random, and the sum must be 1.

**Supplementary Figures**

**
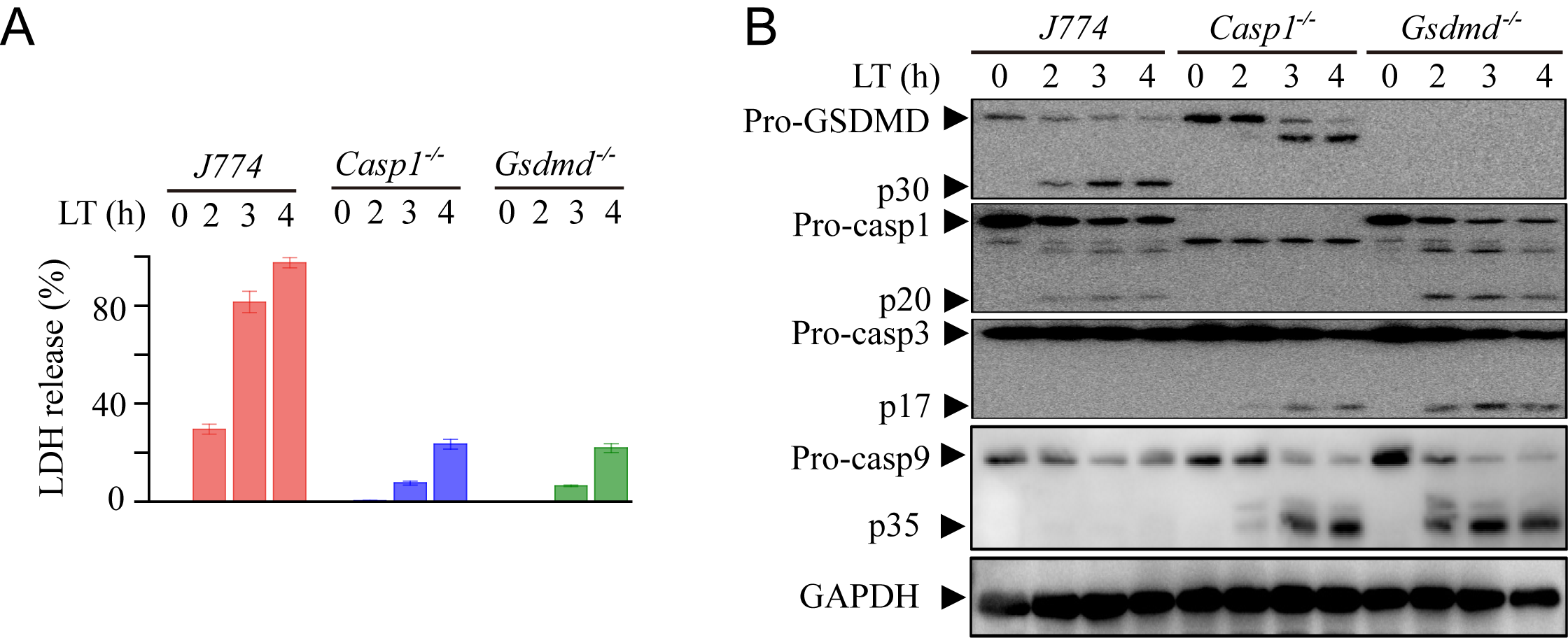
**

**Figure S1. Analysis of *Casp1* or *Gsdmd* knockout in J774A.1 cells. (A)** Effects of genetic deletion of *Casp1* or *Gsdmd* on inflammasome induced LDH release for indicated times. **(B)** Western blot analysis of the effects of genetic deletion of *Casp1* or *Gsdmd* on indicated proteins activation.


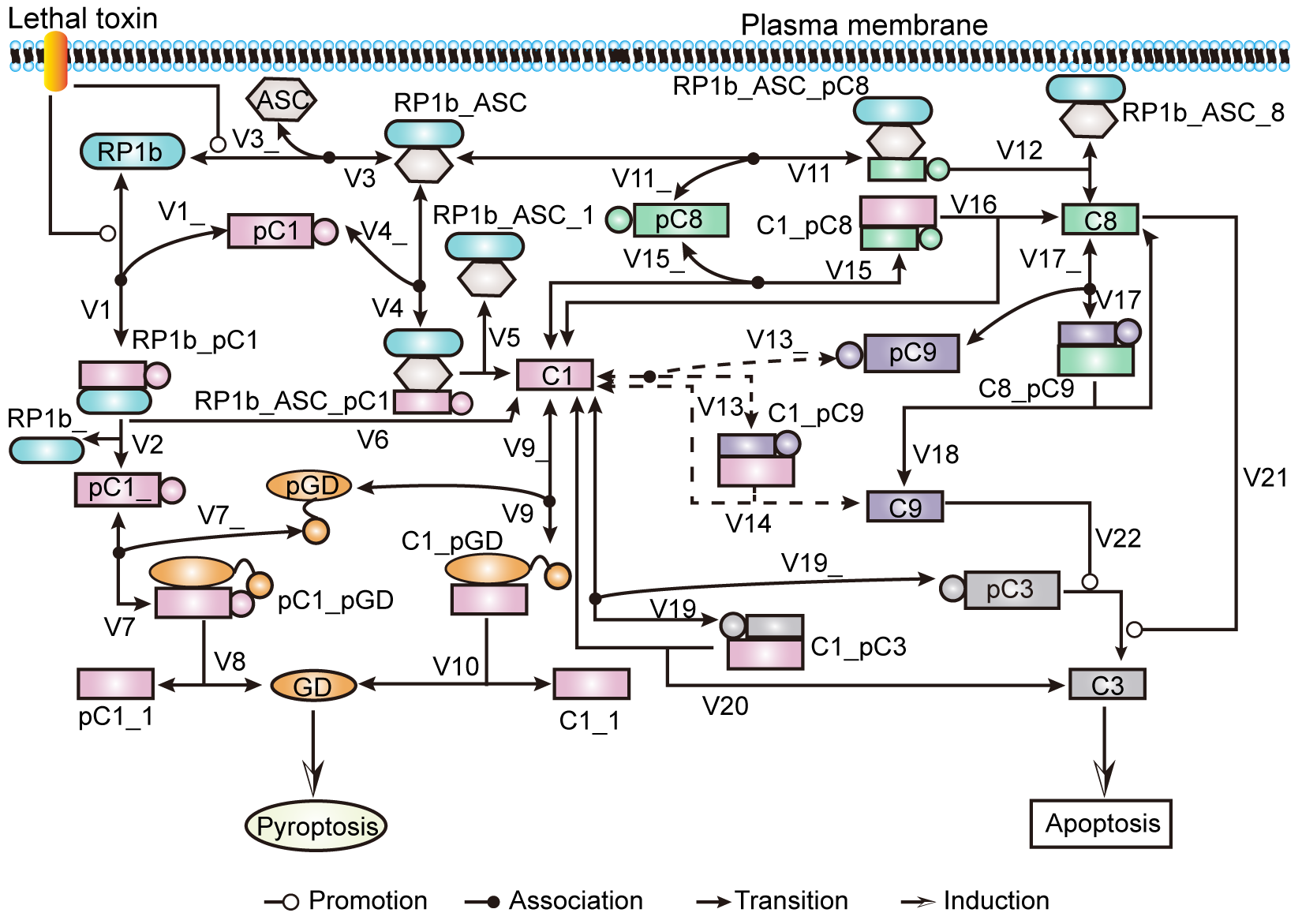


**Figure S2. Kinetic scheme of the NLRP1b inflammasome-induced cell death signaling model.** Lines ended by symbols represent the biochemical reactions characterized by Reactions V1-V22 in Table S1. Initial value of each transducer is shown in Table S2 and parameters for the individually numbered reactions are given in Table S3.


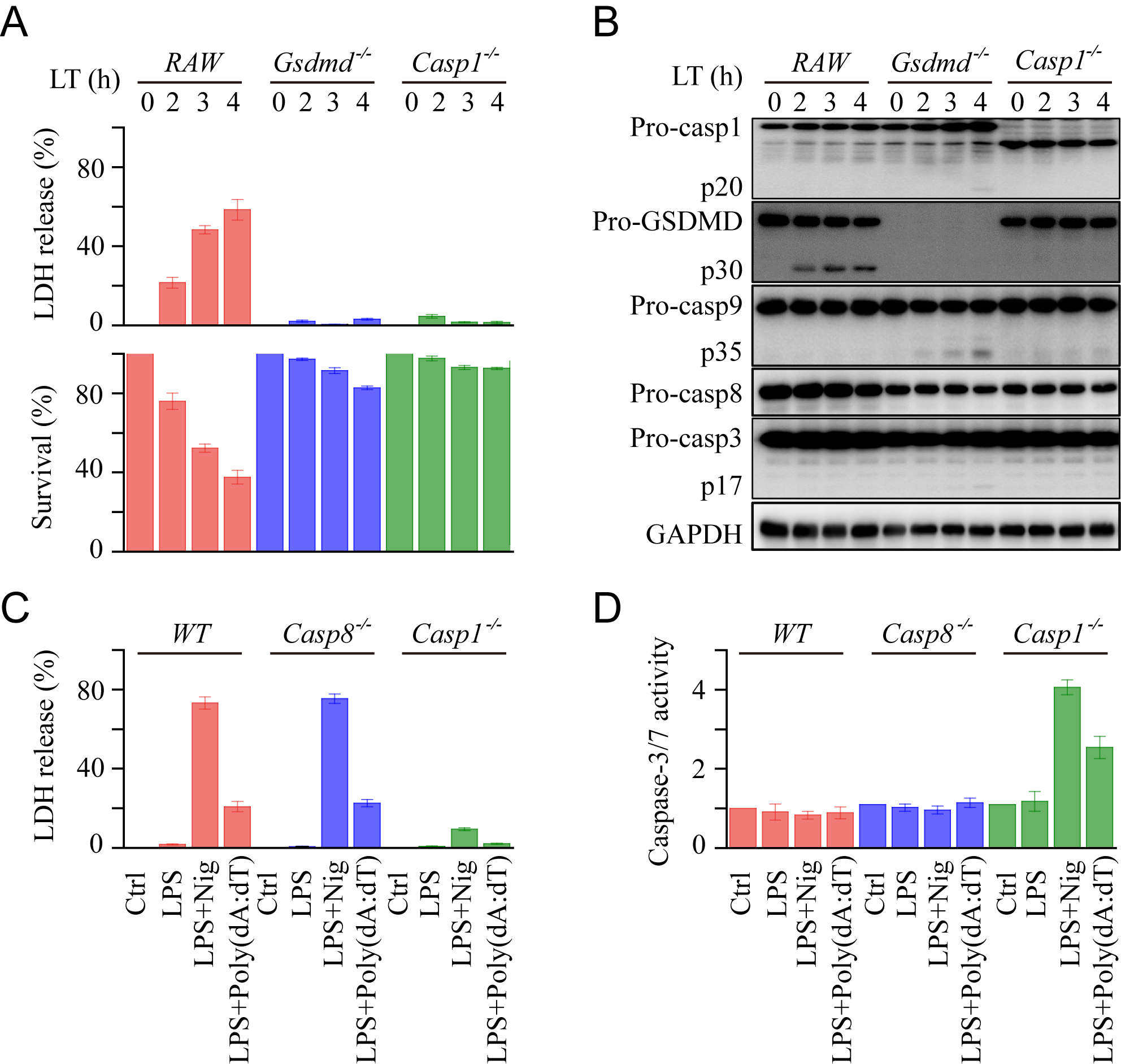


**Figure S3. Analysis of *Casp1*, *Gsdmd* or *Casp8* knockout in regulating cell death. (A)** Effects of genetic deletion of *Casp1* or *Gsdmd* on inflammasome induced LDH release and cell survival for indicated times. **(B)** Western blot analysis of the effects of genetic deletion of *Casp1* or *Gsdmd* on indicated proteins activation. **(C)-(D)** Effects of *Casp8* or *Casp1* deletion on NLRP3 and AIM2 inflammasome-mediated cell death in THP1 cells.

**Supplementary Tables**

**Table S1. Reactions and reaction rates**

| **No.** | **Reactions** | **Reaction rates** |
| --- | --- | --- |
| V1 | N1b + pC1 → N1b_pC1 | $k_{1}*LT^{n_{1}}/(LT^{n_{1}}+j_{1}^{n_{1}})*[N1b\_pC1]$ |
| V1_ | N1b_pC1 → N1b + pC1 | $k_{-1}*[N1b\_pC1]$ |
| V2 | N1b_pC1 + N1b_pC1→ 2 N1b_+2pC1_ | $k_{2}*[N1b\_pC1]*[N1b\_pC1]$ |
| V3 | N1b + ASC → N1b_ASC | $k_{3}*LT^{n_{4}}/(LT^{n_{4}}+j_{4}^{n_{4}})*[N1b]*[ASC]$ |
| V3_ | N1b_ASC →N1b + ASC | $k_{-3}*[N1b\_ASC]$ |
| V4 | N1b_ASC + pC1 → N1b_ASC_pC1 | $k_{4}*[pC1]*[N1b\_ASC]$ |
| V4_ | N1b_ASC_pC1 →N1b_ASC + pC1 | $k_{-4}*[N1b\_ASC\_pC1]$ |
| V5 | N1b_ASC_pC1 + N1b_ASC_pC1→ 2 N1b_ASC+2C1 | $k_{5}*[N1b\_ASC\_pC1]^{2}$ |
| V6 | pC1_ + pC1_ → 2C1 | $k_{6}*[pC1\_]^{2}$ |
| V7 | pC1_+pGD → pC1_pGD | $k_{7}*[pC1\_]*[pGD]$ |
| V7_ | pC1_pGD → pC1_+pGD | $k_{-7}*[pC1\_pGD]$ |
| V8 | pC1_pGD → pC1_ 1+ GD | $k_{8}*[pC1\_pGD]$ |
| V9 | C1+pGD → C1_pGD | $k_{9}*[C1]*[pGD]$ |
| V9_ | C1_pGD→C1+pGD | $k_{-9}*[C1\_pGD]$ |
| V10 | C1_pGD → C1_1+GD | $k_{10}*[C1\_pGD]$ |
| V11 | N1b_ASC + pC8 → N1b_ASC_pC8 | $k_{11}*[N1b\_ASC]*[pC8]$ |
| V11_ | N1b_ASC_pC8 → N1b_ASC + pC8 | $k_{-11}*[N1b\_ASC\_pC8]$ |
| V12 | N1b_ASC_pC8 + N1b_ASC_pC8→ 2 N1b_ASC+2C8 | $k_{12}*[N1b\_ASC\_pC8]^{2}$ |
| V13 | C1 + pC9 → C1_pC9 | $k_{13}*[C1]*[pC9]$ |
| V13_ | C1_pC9 → C1 + pC9 | $k_{-13}*[C1\_pC9]$ |
| V14 | C1_pC9 →C1 + C9 | $k_{14}*[C1\_pC9]$ |
| V15 | C1 + pC8 → C1_pC8 | $k_{15}*[C1]*[pC8]$ |
| V15_ | C1_pC8 → C1 + pC8 | $k_{-15}*[C1\_pC8]$ |
| V16 | C1_pC8 →C1 + C8 | $k_{16}*[C1\_pC8]$ |
| V17 | C8 + pC9 → C8_pC9 | $k_{17}*[C8]*[pC9]$ |
| V17_ | C8_pC9 → C8 + pC9 | $k_{-17}*[C8\_pC9]$ |
| V18 | C8_pC9 →C8 + C9 | $k_{18}*[C1\_p\text{C9}]$ |
| V19 | C1 + pC3 → C1_pC3 | $k_{19}*[C1]*[pC3]$ |
| V19_ | C1_pC3 → C1 + pC3 | $k_{-19}*[C1\_pC3]$ |
| V20 | C1_pC3 →C1+ C3 | $k_{20}*[C1\_p\text{C3}]$ |
| V21 | C8 + pC3 → C8+C3 | $k_{21}*[p\text{C3}]*[\mathrm{C}8]^{n_{2}}/([\mathrm{C}8]^{n_{2}}+j_{2}^{n_{2}})$ |
| V22 | C9 + pC3 → C9+C3 | $k_{22}*[p\text{C3}]*[\mathrm{C}9]^{n_{3}}/([\mathrm{C}9]^{n_{3}}+j_{3}^{n_{3}})$ |

**Table S2. Ordinary differential equations and initial amounts**

| **Components** | **ODEs** | **Initial amount (mpc)** | **References** |
| --- | --- | --- | --- |
| N1b | d[N1b]/dt= -V1+V1_-V3+V3_ | 150000 | [1] |
| pC1 | d[pC1]/dt= -V1+V1_-V4+V4_ | 96000 | [1] |
| N1b_pC1 | d[ N1b_pC1]/dt=V1-V1_-2*V2 | 0 |  |
| pC1_ | d[ pC1_]/dt=V2*2-V6*2-V7+V7_ | 0 |  |
| N1b_ | d[ N1b_]/dt=V2*2 | 0 |  |
| ASC | d[ ASC ]/dt= -V3+V3_ | 3500 | [2, 3] |
| N1b_ASC | d[ N1b_ASC ]/dt=V3-V3_-V4+V4_-V11+V11_ | 0 |  |
| N1b_ASC_pC1 | d[ N1b_ASC_pC1]/dt=V4-V4_-V5*2 | 0 |  |
| N1b_ASC_1 | d[ N1b_ASC_1]/dt=2*V5 | 0 |  |
| C1 | d[ C1]/dt=V5+V6*2-V9+V9_-V13+V13_+V14-V15+V15_+V16-V19+V19_+V20 | 0 |  |
| pGD | d[ pGD ]/dt= -V7+V7_-V9+V9_ | 150000 | [1] |
| pC1_pGD | d[ pC1_pGD ]/dt=V7-V7_-V8 | 0 |  |
| pC1_1 | d[ pC1_1]/dt=V8 | 0 |  |
| GD | d[ GD ]/dt=V8+V10 | 0 |  |
| C1_pGD | d[ C1_pGD ]/dt=V9-V9_-V10 | 0 |  |
| C1_1 | d[ C1_1]/dt=V10 | 0 |  |
| pC8 | d[pC8]/dt= -V11+V11_-V15+V15_ | 7000 | [1] |
| N1b_ASC_pC8 | d[ N1b_ASC_pC8]/dt=V11-V11_-V12*2 |  |  |
| N1b_ASC_8 | d[ N1b_ASC_8]/dt=V12*2 | 0 |  |
| C8 | d[ C8]/dt=V12*2+V16-V17+V17_+V18 | 0 |  |
| pC9 | d[ pC9]/dt= -V13+V13_-V17+V17_ | 7000 | [1] |
| C1_pC9 | d[ C1_pC9]/dt=V13-V13_-V14 | 0 |  |
| C9 | d[C9]/dt=V14+V18 | 0 |  |
| C1_pC8 | d[ C1_pC8]/dt=V15-V15_-V16 | 0 |  |
| C8_pC9 | d[ C8_pC9]/dt=V17-V17_-V18 | 0 |  |
| pC3 | d[ pC3]/dt= -V19+V19_-V21-V22 | 96000 | [1] |
| C1_pC3 | d[ C1_pC3]/dt=V19-V19_-V20 | 0 |  |
| C3 | d[ C3]/dt=-V20+V21+V22 | 0 |  |
| N | d[N]/dt=k_a_*N-k_dC3_*C3*N-k_dGD_*GD *N-k_d_*N*N | 2×10^7^ | Measured |

**Table S3. Parameters of the model**

| **Parameters** | **Description** | **References** |
| --- | --- | --- |
| $\begin{matrix} k_{1}=4.0\times10^{-9}mpc^{-1}s^{-1}, k_{-1}=4\times10^{-3}s^{-1} \\ n_{1}=1, j_{1}=0.3 mpc, LT=1 \mu g/mL \end{matrix}$ | Rates of NLRP1b (N1b) directly binds to pro-caspase-1 (pC1) induced by lethal toxin | Fitted and [4] |
| $k_{2}=9.87\times10^{-6}mpc^{-1}s^{-1}$ | Dimerization rate of N1b_pC1 complex to activate pC1 | Fitted |
| $\begin{matrix} k_{3}=2.386\times10^{-9}mpc^{-1}s^{-1}, k_{-3}=10^{-3}s^{-1} \\ n_{4}=1, j_{4}=0.3 mpc \end{matrix}$ | Rates of N1b binds to ASC induced by lethal toxin | Fitted |
| $k_{4}=2.498\times10^{-4}mpc^{-1}s^{-1}, k_{-4}=1\times10^{-5}s^{-1}$ | Rates of N1b_ASC complex binds to pC1 | Fitted |
| $k_{5}=1.0\times10^{-6}mpc^{-1}s^{-1}$ | Dimerization rate of N1b_ASC_pC1 complex to cleave pC1 | Fitted |
| $k_{6}=2.62\times10^{-9}mpc^{-1}s^{-1}$ | Autoproteolysis rate of pC1_ by dimerization | Estimated |
| $k_{7}=1\times10^{-4}mpc^{-1}s^{-1}, k_{-7}=2\times10^{-3}s^{-1}$ | Rates of pC1_ binds to pro-GSDMD (pGD) | Fitted |
| $k_{8}=5\times10^{-3}s^{-1}$ | Rate of N-terminal of pGD (GD) drops from pC1_pGD complex | Estimated and [5] |
| $k_{9}=8\times10^{-5}mpc^{-1}s^{-1}, k_{-9}=1\times10^{-4}s^{-1}$ | Rates of cleaved caspase-1 (C1) binds to pGD | Fitted |
| $k_{10}=8\times10^{-4}s^{-1}$ | Rate of GD drops from C1_pGD complex | Estimated |
| $k_{11}=8.75\times10^{-8}mpc^{-1}s^{-1} ,k_{-11}=2\times10^{-5}s^{-1}$ | Rates of N1b_ASC complex binds to pro-caspase-8 (pC8) | Fitted |
| $k_{12}=4.64\times10^{-7}mpc^{-1}s^{-1}$ | Dimerization rate of N1b_ASC_pC8 complex to cleave pC8 | Fitted and [6, 7] |
| $k_{13}=3\times10^{-6}mpc^{-1}s^{-1}, k_{-13}=1\times10^{-3}s^{-1}$ | Rates of C1 binds to pro-caspase-9 (pC9) | Estimated and [7] |
| $k_{14}=2.362\times10^{-4}s^{-1}$ | Rate of cleaved caspase-9 (C9) drops from C1_pC9 complex | Fitted |
| $k_{15}=5.67\times10^{-7}mpc^{-1}s^{-1}, k_{-15}=0.001s^{-1}$ | Rates of C1 binds to pro-caspase-8 (pC8) | Estimated |
| $k_{16}=2.244\times10^{-4}s^{-1}$ | Rate of cleaved caspase-8 (C8) drops from C1_pC8 complex | Fitted |
| $k_{17}=3.3\times10^{-7}mpc^{-1}s^{-1}, k_{-17}=1\times10^{-3}s^{-1}$ | Rates of C8 binds to pC9 | [8] |
| $k_{18}=8.08\times10^{-4}s^{-1}$ | Rate of C9 drops from C8_pC9 complex | Fitted |
| $k_{19}=1.286\times10^{-9}mpc^{-1}s^{-1}$ | Rates of C1 binds to pro-caspase-3 (pC3) | Fitted and [9] |
| $k_{20}=2.533\times10^{-4}s^{-1}$ | Rate of cleaved caspase-3 (C3) drops from C1_pC3 complex | Fitted |
| $k_{21}=1.558\times10^{-4}s^{-1}, n_{2}=7, j_{2}=725mpc$ | Rate and michaelis constant of C8 activate pC3 | [10, 11] |
| $k_{22}=1\times10^{-9}s^{-1}, n_{3}=15, j_{2}=2\times10^{4}mpc$ | Rate and michaelis constant of C9 activate pC3 | [10, 12] |
| N | Population of cells | Measured |
| $k_{a}=3.4\times10^{-6}s^{-1}, k_{d}=1.7\times10^{-13}mpc^{-1}s^{-1}$ | Rate of basal cell proliferation and death | Fitted |
| $k_{dC3}=4\times10^{-9}s^{-1}, k_{dGD}=1.732\times10^{-8}s^{-1}$ | Cell death rate of apoptosis and pyroptosis | Fitted |

References

[1] B. Schwanhaeusser, D. Busse, N. Li et al., "Global quantification of mammalian gene expression control," *Nature*, vol. 473, no. 7347, pp. 337-342, 2011.

[2] M. S. Dick, Sborgi L, Ruhl S, Hiller S, and Broz P, "ASC filament formation serves as a signal amplification mechanism for inflammasomes," *Nature Communications*, vol. 7, article 11929, 2016.

[3] P. R. Vajjhala, A. Lu, D. L. Brown et al., "The Inflammasome Adaptor ASC Induces Procaspase-8 Death Effector Domain Filaments," *Journal of Biological Chemistry*, vol. 290, no. 49, pp. 29217-29230, 2015.

[4] N. Van Opdenbosch, P. Gurung, L. Vande Walle et al., "Activation of the NLRP1b inflammasome independently of ASC-mediated caspase-1 autoproteolysis and speck formation," *Nature Communications*, vol. 5, article 3209, 2014.

[5] W. He, H. Wan, L. Hu et al., "Gasdermin D is an executor of pyroptosis and required for interleukin-1 beta secretion," *Cell Research*, vol. 25, no. 12, pp. 1285-1298, 2015.

[6] N. Van Opdenbosch, H. Van Gorp, M. Verdonckt et al., "Caspase-1 Engagement and TLR-Induced c-FLIP Expression Suppress ASC/Caspase-8-Dependent Apoptosis by Inflammasome Sensors NLRP1 band NLRC4," *Cell Reports*, vol. 21, no. 12, pp. 3427-3444, 2017.

[7] K. Tsuchiya, S. Nakajima, S. Hosojima et al., "Caspase-1 initiates apoptosis in the absence of gasdermin D," *Nature Communications*, vol. 10, article 2091, 2019.

[8] P. Zhang, Y. Liu, L. Hu et al., "NLRC4 inflammasome-dependent cell death occurs by a complementary series of three death pathways and determines lethality in mice," *Science Advances*, vol. 7, no. 43, article eabi9471, 2021.

[9] C. Y. Taabazuing, M. C. Okondo, and D. A. Bachovchin. "Pyroptosis and Apoptosis Pathways Engage in Bidirectional Crosstalk in Monocytes and Macrophages," *Cell Chemical Biology*, vol. 24, no. 4, pp. 507-514, 2017.

[10] S. Legewie, N. Bluthgen, and H. Herzel, "Mathematical modeling identifies inhibitors of apoptosis as mediators of positive feedback and bistability," *Plos Computational Biology*, vol. 2, no. 9, pp. 1061-1073, 2006.

[11] M. W. Anderson, J. J. Moss, R. Szalai, and J. D. Lane, "Mathematical Modeling Highlights the Complex Role of AKT in TRAIL-Induced Apoptosis of Colorectal Carcinoma Cells," *iScience*, vol. 12, pp.182-193, 2019.

[12] J. G. Albeck, J. M. Burke, S. L. Spencer, D. A. Lauffenburger, and P. K. Sorger, "Modeling a Snap-Action, Variable-Delay Switch Controlling Extrinsic Cell Death," *Plos Biology*, vol. 6, no. 12, pp. 2831-2852, 2008.
